# Metabolite Profiling of Rambutan (*Nephelium lappaceum* L.) Seeds Using UPLC-qTOF-MS/MS and Senomorphic Effects in Aged Human Dermal Fibroblasts

**DOI:** 10.3390/nu12051430

**Published:** 2020-05-15

**Authors:** Yae Rin Lee, Hyo Moon Cho, Eun Jin Park, Mi Zhang, Thi Phuong Doan, Ba Wool Lee, Kyung A Cho, Won Keun Oh

**Affiliations:** 1 Korea Bioactive Natural Material Bank, Research Institute of Pharmaceutical Sciences, College of Pharmacy, Seoul National University, Seoul 08826, Korea; leeyaerin93@snu.ac.kr (Y.R.L.); chgyans@naver.com (H.M.C.); eunjin_p@snu.ac.kr (E.J.P.); mintazhang@snu.ac.kr (M.Z.); phuongdoan@snu.ac.kr (T.P.D.); paul36@snu.ac.kr (B.W.L.); 2 Department of Biochemistry, Chonnam National University Medical School, Gwangju 51828, Korea; kacho@jnu.ac.kr

**Keywords:** *Nephelium lappaceum*, cellular senescence, UPLC-qTOF-MS, senescence-associated secretory phenotype (SASP), senomorphic, human dermal fibroblast

## Abstract

*Nephelium lappaceum* (rambutan) is an edible tropical fruit that is widely grown in Southeast Asia. In general, the seeds contain high nutrients, but rambutan seeds are thrown out during processing. In this study, the anti-aging activity of rambutan seeds was evaluated with a new approach through the selective inhibition of the senescence-associated secretory phenotype (senomorphics). Luciferase promoter assays using p16INK4A and SA-β-gal promoters for rambutan showed that its seeds possessed strong senomorphic activity. Molecular networking using ultra-performance liquid chromatography-quadrupole time-of-flight mass spectrometry (UPLC-qTOF-MS) with a tandem database (UPLC-qTOF-MS/MS) was applied to determine the chemical composition of rambutan. Based on the activity results, nine compounds, one new (**7**) and eight known kaempferol type compounds, were isolated from the seeds. Compounds **2**, **4** and **9** significantly reduced the mRNA expression levels of senescence markers, such as p16INK4A, p21CIP1, p53 and SA-β-gal. These compounds also significantly increased the level of SIRT1, a longevity modulator. Compounds **2**, **4** and **9** decreased the mRNA expression levels of senescence-associated secretory phenotypes (SASPs) and subsequently decreased the number of SA-β-gal-positive cells. Thus, rambutan seeds and its constituents might be able to protect against age-related problems.

## 1. Introduction

Aging is the progressive decrease in the ability of tissues to recover from stress and is associated with physical dysfunction, incapacity and considerable morbidity, and these factors increase the burden of age-related chronic diseases. Nearly 92% of older adults have at least one chronic disease, and 77% have at least two [1]. Thus, it is imperative to find a way to therapeutically target the process of aging to increase the healthspan as well as remaining lifespan in old age. In the last 10 years, groundbreaking studies have provided proof of the concept that either relieving the burden of senescent cells or suppressing the senescence-associated secretory phenotype (SASP) can have a beneficial impact on age-related abnormalities, thus ameliorating the health of an organism and subsequently increasing its life span. In this direction, the discovery that small molecules can act as selective eliminators of senescent cells (senolytics) or as inhibitors of the SASP (senomorphics) has paved the way to an exciting new field of research aimed at the development of senotherapeutics [2]. The first molecules to be reported as senolytics in 2015 were dasatinib, a clinically used kinase inhibitor, and quercetin, a common flavonoid [3]. The potential activity of BCL-2 inhibitors (Navitoclax, ABT-263), flavonols, fisetin and the alkaloid piperlongumine against senescent cells was subsequently established [4].

Cellular senescence, a key mechanism that has been demonstrated to drive aging, is a state of irreversible cell cycle arrest that occurs as a result of different stresses or stimuli, such as telomere shortening, oxidative stress, DNA damage or the aberrant activation of oncogenes [5]. Senescent cells have an enlarged and flattened shapes with slower division and elevated SA-β-gal activity [6], which remains the gold standard for identifying senescent cells in culture and tissue samples and the increased expression of the cyclin-dependent kinase inhibitors (CDKIs) p16INK4A and p21 [7]. In particular, p16INK4A plays an important role in cell cycle arrest, and it is upstream of the retinoblastoma tumor suppressor protein and accumulates in an age-dependent manner in various tissues, including the skin. SA-β-gal, expressed from the GLB1 gene, is the most widely used biomarker for senescent cells due to the increased expression of the lysosomal β-galactosidase protein.

Senescent cells also secrete a number of soluble factors associated with growth factors and a pro-inflammatory cytokine response known as SASP, including IL-1, IL-6, IL-8, proteases and matrix metalloproteinases [8]. Recent data have demonstrated that the activation or enforced expression of sirtuins (SIRTs) increases lifespan in animal models, making sirtuins a potential target for healthy aging. Silent mating type information regulation 2 homolog (SIRT1), also known as NAD-dependent deacetylase-1, also mediates the beneficial antiaging effects of caloric restriction and natural products, such as resveratrol, which can extend the human lifespan [9].

Phytochemicals are being increasingly recognized in the field of healthy aging as promising therapeutics against a number of aging-related diseases. *Nephelium lappaceum* L., commonly known as rambutan, belongs to the genus *Nephelium* and is commonly grown in Southeast Asia [10]. Its fruit is a commercially important crop in Asia and it is consumed fresh, canned or processed. In Malaysia, its dried fruit peels have been employed in local medicine [11]. In previous studies, the antioxidant and phenolic contents of the peel and seeds from the fruits of rambutan were evaluated. Butylated hydroxytoluene (BHT), gallic acid, ellagic acid, corilagin and geraniin were isolated from *N. lappaceum* L. peel, and the antioxidant activities of these compounds were determined through lipid peroxidation inhibition and DPPH radical scavenging assays [10]. Few studies on the identification of phenolic compounds in seeds from the genus *Nephelium* have been published. Dereplication is crucial to discovering the potential bioactive compounds and prevent the re-isolation of known compounds. Based on ultra-performance liquid chromatography-quadrupole time-of-flight mass spectrometry (UPLC-qTOF-MS) with a tandem database, Global Natural Product Social Molecular Networking (GNPS) was applied for the dereplication of the chemical components in the fruit of *N. lappaceum*. One new compound (**7**) and eight known kaempferol type compounds (**1**–**6**, **8** and **9**) were obtained from the seeds of this fruit through bioactivity-guided fractionation. In this study, phenolic compounds **2**, **4** and **9** were selected as candidates to suppress senescence and other aging-related diseases. Their effects on cellular senescence in human dermal fibroblasts (HDFs) were examined, and the underlying biological mechanism was examined by focusing on senescence-associated markers and the secretory phenotype, especially p16INK4A, SA-β-gal, sirtuin 1 (SIRT1) and SASP.

## 2. Materials and Methods

### 2.1. Experimental Instruments

Optical rotation values were measured on a JASCO P-2000 polarimeter (JASCO, Easton, MD, USA). UV spectra were recorded on a Chirascan-Plus CD spectrometer (Applied Photophysics, Leatherhead, UK). IR spectra were recorded on a JASCO FT/IR-4200 spectrometer. 1D and 2D NMR data were obtained on Bruker Advance 400, 500 (Bruker, Rheinstetten, Germany) and JNM-ECA-600 (JEOL Ltd., USA) spectrometers at the College of Pharmacy, Seoul National University, Korea. HRESIMS spectra were obtained with a Waters Xevo G2 qTOF mass spectrometer (Waters Co., Milford, MA, USA). Open column chromatography separations were carried out using Sephadex LH-20 (Sigma-Aldrich, St. Louis, MO, USA). TLC analyses were performed with TLC silica gel 60 RP-C18 F254S plates (Merck, Darmstadt, Germany). Preparative HPLC separations were performed using a Gilson system (Gilson, Inc., Villiers-le-Bel, France) with a UV detector at 201 or 254 nm and an Optima Pak C18 column (10 × 250 mm, 5 μm, RS Tech, Seoul, Korea) or a YMC-Triart phenyl column (10 × 250 mm, 5 μm, YMC Co., Ltd., Kyoto, Japan). All the solvents used for extraction and isolation were of analytical grade.

### 2.2. Plant Material

*Nephelium lappaceum* fruit (Rambutan) was purchased from a supermarket in Seoul, South Korea, in May 2019. Professor Won Keun Oh botanically identified the sample. A voucher specimen (SNU 2019-17) was deposited at the College of Pharmacy, Seoul National University, Seoul, Korea.

### 2.3. UHPLC-qTOF-MS^2^ Experiments

The chemical profiling of *N. lappaceum* was performed using a Xevo G2 qTOF mass spectrometer. A Waters Acquity UHPLC^®^ BEH C18 (100 mm × 2.1 mm, 1.7 μm) column was used for the chromatographic analysis and the column temperature was maintained at 40 °C. LC-MS/MS analyses were performed using fast data-dependent acquisition (DDA) mode. The chromatographic separation was performed using a linear gradient of H_2_O (with 0.1% aqueous formic acid, A) and acetonitrile (with 0.1% aqueous formic acid, B) as follows: 0–7 min, 10%–90% B; and 7.1–8 min, 100% B. The ESI conditions were set to the following parameters: an untargeted MS scan from 50 to 1500, negative ion mode, a source temperature of 120 °C, a capillary voltage of 1.5 kV, a cone voltage of 40.0 V, a cone gas flow rate of 50.0 L/h, a desolvation gas flow rate of 800 L/h, a cone gas flow rate of 50.0 L/h and a collision energy of 20 to 40 eV.

### 2.4. Sample Preparation of Nephelium lappaceum for MS/MS Analysis

A fresh whole rambutan (33.1 g) was separated into peels (25.2 g), pulp (3.1 g) and seeds (4.8 g), and each sample (3.0 g) was extracted by sonication for 2 h with 20 mL of 70% EtOH at room temperature. The dried 70% EtOH extracts were then suspended in distilled water and sequentially partitioned into *n*-hexane, ethyl acetate (EA), *n*-BuOH and water. All the samples were dried and diluted to a final concentration of 1.0 mg/mL with HPLC-grade MeOH for analysis. These solutions were passed through disposable 0.20 μm membrane filters (Advantec, Tokyo Roshi Kaisha, Japan), and 1.0 μL of each sample was injected and analyzed.

### 2.5. Extraction and Isolation of Flavonoid Glycosides

The fresh seeds of *N. lappaceum* (1.2 kg) were extracted with 70% EtOH at room temperature, and the filtered extract was concentrated in vacuo to yield 93.1 g of residue. The dried crude extract was sequentially partitioned between the *n*-hexane (10.3 g), EA (9.8 g), *n*-BuOH (25.8) and water (46.7 g). A precipitate was obtained from the ethyl acetate-soluble fraction, and this precipitate was purified by semipreparative HPLC to obtain compound **9** (50.0 mg). The *n*-BuOH-soluble fraction was initially subjected to Sephadex LH-20 column chromatography eluting with 100% MeOH to yield subfractions Bu1 (14.0 g), Bu2 (6.0 g) and Bu3 (5.8 g). Obtained fraction Bu1 was purified on an Optima Pak C18 column eluting with 20% MeOH/H_2_O containing 0.1% formic acid by semipreparative HPLC to obtain compounds **5** (15.0 mg), **8** (18.1 mg), and **7** (8.0 mg). The obtained fraction Bu2 was purified by semipreparative HPLC using an Optima Pak C18 column with 35% MeOH/H_2_O to yield subfraction Bu2.1 (15 mg) and compounds **1** (6.0 mg), **2** (15.0 mg), and **3** (4.0 mg). Subtraction Bu2.1 was further purified by semipreparative HPLC eluting with 20% MeOH/H_2_O containing 0.1% formic acid on a YMC-Triart phenyl column to obtain compound **4** (4.2 mg). Compound **6** (4.8 mg) was isolated from subfraction Bu3 by semipreparative HPLC using an Optima Pak C18 column eluting with 45% MeOH/H_2_O containing 0.1% formic acid.

Kaempferol 3-O-β-d-galactopyranosyl-7-O-α-l-rhamnopyranoside (**1**): Yellow, amorphous powder; ${\left[\alpha \right]}_{D}^{20}$ −40.4 (*c* 0.1, MeOH); UV (MeOH) *λ*_max_ (log *ε*) 200 (4.76), 265 (4.40), 350 (4.17) nm; IR *ν*_max_ 3390, 1648, 1488, 1355, 1268, 1205, 1060, 993, 891 cm^−1^; ^1^H and ^13^C NMR data, see Figures S1–S4, Tables S1 and S2 in Supplementary Materials; HRESIMS *m*/*z* 593.1505 [M − H]^─^ (calcd for C_27_H_29_O_15_, 593.1506).

Kaempferol 3-O-β-d-glucopyranosyl-7-O-α-l-rhamnopyranoside (**2**): Yellow, amorphous powder; ${\left[\alpha \right]}_{D}^{20}$ −134.8 (*c* 0.1, MeOH); UV (MeOH) *λ*_max_ (log *ε*) 200 (4.29), 265 (4.04), 350 (3.78) nm; IR *ν*_max_ 3389, 2925, 1661, 1495, 1310, 1208, 1078, 811 cm^−1^; ^1^H and ^13^C NMR data, see Figures S5–S8, Tables S1 and S2 in Supplementary Materials; HRESIMS *m*/*z* 593.1489 [M − H]^─^ (calcd for C_27_H_29_O_15_, 593.1506).

Kaempferol-3-O-α-l-arabinopyranosyl-7-O-α-l-rhamnopyranoside (**3**): Yellow, amorphous powder; ${\left[\alpha \right]}_{D}^{20}$ −51.6 (*c* 0.1, MeOH); UV (MeOH) *λ*_max_ (log *ε*) 200 (4.19), 265 (3.87), 350 (3.65) nm; IR *ν*_max_ 3325, 2938, 1685, 1174, 1108, 1054, 810 cm^−1^; ^1^H and ^13^C NMR data, see Figures S9–S12, Tables S1 and S2 in Supplementary Materials; HRESIMS *m*/*z* 563.1376 [M − H] ^─^ (calcd for C_26_H_27_O_14_, 563.1401).

Kaempferol 3-O-rutinoside (**4**): Yellow, amorphous powder; ${\left[\alpha \right]}_{D}^{20}$ −81.8 (*c* 0.1, MeOH); UV (MeOH) *λ*_max_ (log *ε*) 200 (4.32), 265 (3.93), 350 (3.73) nm; IR *ν*_max_ 3385, 2920, 1657, 1508, 1362, 1181, 1024, 840 cm^−1^; ^1^H and ^13^C NMR data, see Figures S13–S16, Tables S1 and S2 in Supplementary Materials; HRESIMS *m*/*z* 593.1525 [M − H]^─^ (calcd for C_27_H_29_O_15_, 593.1506).

Ternatumoside X (**5**): Yellow, gummy solid; ${\left[\alpha \right]}_{D}^{20}$ −154.4 (*c* 0.1, MeOH); UV (MeOH) *λ*_max_ (log *ε*) 200 (4.83), 225 (4.45), 265 (4.47), 350 (4.57) nm; IR *ν*_max_ 3393, 2937, 1657, 1603, 1514, 1348, 1173, 1032, 832 cm^−1^; ^1^H and ^13^C NMR data, see Figures S17–S20, Tables S1 and S2 in Supplementary Materials; HRESIMS *m*/*z* 1047.2950 [M − H]^─^ (calcd for C_48_H_55_O_26_, 1047.2982).

Astragalin (**6**): Yellow, amorphous powder; ${\left[\alpha \right]}_{D}^{20}$ −112.7 (*c* 0.1, MeOH); UV (MeOH) *λ*_max_ (log *ε*) 200 (4.24), 265 (3.78), 370 (3.66) nm; IR *ν*_max_ 3393, 1657, 1608, 1508, 1362, 1181, 1024, 828 cm^−1^; ^1^H and ^13^C NMR data, see Figures S21–S24, Tables S1 and S2 in Supplementary Materials; HRESIMS *m*/*z* 447.0919 [M − H]^─^ (calcd for C_21_H_19_O_11_, 447.0927).

Compound **7** (**7**): Pale-yellow, gummy solid; ${\left[\alpha \right]}_{D}^{20}$ −83.3 (*c* 0.1, MeOH); UV (MeOH) *λ*_max_ (log *ε*) 200 (4.20), 265 (3.79), 370 (3.75) nm; IR *ν*_max_ 3373, 1655, 1605, 1514, 1353, 1175, 1024, 828 cm^−1^; ^1^H and ^13^C NMR data, see Figures S25–S31, Table S3 in Supplementary Materials; HRESIMS *m*/*z* 885.2432 [M − H]^─^ (calcd for C_42_H_45_O_21_, 885.2453).

5-hydroxy-2-(4-hydroxyphenyl)-4-oxo-7-[(α-l-rhamnopyranosyl)oxy]-4H-chromen-3-yl [6-O-[(2E)-3-(4-hydroxyphenyl)prop-2-enoyl]-β-d-glucopyranosyl-(1→2)]-[β-d-glucopyranosyl-(1→4)]-[6-O-[(2E)-3-(4-hydroxyphenyl)prop-2-enoyl]-β-d-glucopyranosyl-(1→3)]-α-l-rhamnopyranoside (**8**): Yellow, gummy solid; ${\left[\alpha \right]}_{D}^{20}$ −130.8 (*c* 0.1, MeOH); UV (MeOH) *λ*_max_ (log *ε*) 200 (4.13), 225 (3.94), 270 (3.79), 350 (3.80) nm; IR *ν*_max_ 3371, 1604, 1514, 1350, 1262, 1172, 1080, 832 cm^−1^; ^1^H and ^13^C NMR data, see Figures S32–S35, Tables S1 and S2 in Supplementary Materials; HRESIMS *m*/*z* 1355.3875 [M − H]^─^ (calcd for C_63_H_41_O_33_, 1355.3878).

Kaempferol 7-O-α-l-rhamnopyranoside (**9**): Pale-yellow, amorphous powder; ${\left[\alpha \right]}_{D}^{20}$ −29.0 (*c* 0.1, MeOH); UV (MeOH) *λ*_max_ (log *ε*) 200 (4.38), 265 (3.95), 370 (3.84) nm; IR *ν*_max_ 3383, 2921, 1657, 1597, 1489, 1351, 1287, 1207, 1180, 1061, 1011, 824 cm^−1^; ^1^H and ^13^C NMR data, see Figures S36–S39, Tables S1 and S2 in Supplementary Materials; HRESIMS *m*/*z* 431.0976 [M − H]^─^ (calcd for C_21_H_19_O_10_, 431.0978).

### 2.6. Sugar Analysis

The absolute configurations of the monosaccharides were determined according to the method described in previous papers from the same laboratory (Figure S40) [12].

### 2.7. Cloning of the Human p16INK4A and GLB1 Promoter in a Reporter Plasmid

To generate a reporter construct of the human p16INK4A promoter (GenBank Accession No. NM_000077.4), a 420 bp DNA fragment including the promoter of the human p16INK4A gene (-722 to -180) was PCR-amplified using genomic DNA from 293T as a template, nPfu-Forte DNA polymerase (Enzynomics, Daejeon, Korea), and the primer pair 5′-CCCGGTACCGTGGAAGAAAAGGGGAGGAG-3′, which included a KpnI site and 5′-CCCCTCGAGCCGGACTAGGTAGGTGGAGTC-3′, which included an XhoI site. The PCR product cleaved with KpnI and XhoI was cloned into the pGL3-basic luciferase reporter (Promega, Madison, WI, USA) and digested with KpnI and XhoI to generate the pGL3_p16INK4A promoter. The reporter construct of the human GLB1 promoter (GenBank Accession No. NM_000404.4) consisted of a 466 bp DNA fragment including the promoter of the human GLB1 gene. Genomic DNA from 293T as a template was PCR-amplified with nPfu-Forte DNA polymerase (Enzynomics, Daejeon, Korea). The primer pairs of 5′-CCCGGTACCCCGTATATGAGACGCGGATT-3′, which included a KpnI site, and 5′-CCCCTCGAGCAGCAGAACCAGCAACAGAG-3′, which included an XhoI site, were used. The PCR product cleaved with KpnI and XhoI was cloned into the pGL3-basic luciferase reporter and digested with KpnI and XhoI to generate the pGL3_GLB1 promoter. The recombinants were then transformed into *Escherichia coli* and confirmed by DNA sequencing.

### 2.8. Cell Culture

A primary culture of human dermal fibroblasts (HDFs) was obtained from Chonnam National University. The cells were maintained under subconfluent conditions in Dulbecco’s modified Eagle’s medium (DMEM; HyClone, Logan, UT, USA) supplemented with 10% fetal bovine serum (FBS; HyClone) and 1% penicillin/streptomycin (P/S; HyClone) at 37 °C in an atmosphere of 5% CO2 and 95% air.

### 2.9. Cell Viability Assay

The cell viability was assessed using an MTT (3-(4,5-dimethylthiazol-2-yl)-2,5-diphenyltetrazolium bromide)-based cytotoxicity assay. The HDF cells were seeded at a density of 5 × 10^3^ cells/well in 96-well plates and allowed to adhere for 24 h prior to the treatment with the test compounds. The cells were treated with various concentrations of the test compounds in 96-well plates and incubated for 72 h. The final concentration of DMSO in the culture medium was maintained at 0.05% to prevent solvent toxicity. Subsequently, 20 μL of a 2 mg/mL MTT solution was added to each well of the plate, and the cells were incubated for 2 h. Then, the absorbance was measured at 570 nm using a Versamax microplate reader. The percent cell viability was inversely proportional to the toxicity of the compounds, meaning that a higher toxicity corresponded to lower cell viability. The cell viability was defined as the absorbance in the experimental well compared to that in the DMSO control wells.

### 2.10. Luciferase Reporter Assay

The transfection of the reporter genes into the HDFs was conducted using a Lipofectamine LTX (Thermo Fisher Scientific). After transfection with the reporter gene, the transfection efficiency was measured with a GFP-tagged plasmid. The HDFs (3 × 10^4^ cells/well) were seeded in 24-well plates, and the medium was replaced with fresh DMEM supplemented with 10% FBS. The pGL3_p16INK4A (or pGL3_GLB1) promoter (0.4 μg) encoding firefly luciferase driven by the p16INK4A (or GLB1) promoter and β-galactosidase (0.1 μg, encoding Renilla luciferase in 25 μL Opti-MEM) were incubated with the Lipofectamine LTX (0.75 μL, Invitrogen, Carlsbad, CA, USA) and PLUSTM reagent (0.25 μL, Invitrogen, Carlsbad, CA, USA) in 25 μL of Opti-MEM (Gibco/Thermo Fisher Scientific, Waltham, MA, USA) for 10 min at room temperature. To each well was added 50 μL of the DNA-lipid complex. The cells were treated with this mixture for 5 h and then incubated with DMEM supplemented with 10% FBS for 24 h. The cells were then treated with the test compound or DMSO in a serum-free medium for 24 h. The luciferase activity was measured using the luciferase reporter assay system (Promega), and the firefly luciferase activity in the transfected cells was normalized to the Renilla luciferase activity.

### 2.11. Quantitative Reverse Transcription-Polymerase Chain Reaction

Senescent human dermal fibroblasts were simultaneously treated with the candidate compounds, and the qRT-PCR analysis was carried out to measure the levels of cell cycle arrest genes and SASP mRNA. The total RNA was extracted using a TRIzol reagent (Thermo Fisher Scientific, Waltham, MA, USA) according to the manufacturer’s instructions, and the cDNA was synthesized with M-MLV reverse transcriptase (Invitrogen). Quantitative PCR was carried out with a SYBR qPCR mix (Bioneer Co., Korea). The levels of cell cycle arrest genes and SASP mRNAs were normalized to 18S rRNA. Real-time PCR was conducted using the StepOnePlus Real-time PCR system (Applied Biosystems, Inc., Foster City, CA, USA). The data were analyzed with the StepOne software v2.3. The senescence-associated genes and SASP primers for the human p16INK4A, p53, p21, SA-β-gal, IL-6, IL-8, IL-1α, IL-1β and SIRT1 are shown in Table S4 of Supplementary Materials.

### 2.12. Senescence-Associated β-Galactosidase Activity

Using an SA β-galactosidase staining kit (at# 9860; Cell Signaling Technology, Denver, MA, USA), the SA-β-gal-positive cells were stained according to the manufacturer’s instructions. Cells stained for SA-β-gal activity were observed and imaged using a fluorescence microscope (Olympus ix70, Olympus Corporation, Tokyo, Japan).

### 2.13. Statistical Analysis

The data were calculated as the means ± standard deviations (SDs) of three independent experiments. The differences between the means of the two groups were determined by one-way analysis of variance (ANOVA) followed by Tukey’s, Dunnett’s or Duncan’s post hoc tests (SPSS Statistics 23, Chicago, IL, USA). Statistical significance was accepted at * *p* < 0.05, ** *p* < 0.01, and *** *p* < 0.001.

## 3. Results

### 3.1. Molecular Networking of the Seed, Pulp and Peel Extracts of N. lappaceum Fruits

Based on the p16INK4A and SA-β-gal luciferase reporter gene assays, unlike the pulp and peel extracts, the *N. lappaceum* seed extract significantly reduced the luciferase activity (by approximately 70% relative to the control) driven by the p16INK4A and SA-β-gal promoters (Figure S42 in Supplementary Materials). The molecular network (MN) was visualized by MS/MS data, which confirmed the relationship between the senescence-associated promoter activity and the different parts of the *N. lappaceum* extract. In the generated MN, four main clusters, including green nodes, red nodes and blue nodes, were found in the extracts of the *N. lappaceum* seeds, pulp and peels (Figure S43 in Supplementary Materials). The main chemical components in the three different *N. lappaceum* extracts were identified by comparing the references with the MS fragmentation pattern of each peak (Figure S44 in Supplementary Material). Specifically, the DAD chromatogram and the MS/MS fragmentation profiles showed that the pulp of *N. lappaceum* mainly contained disaccharides, while the major components of the *N. lappaceum* peels were identified as gallic acid, corilagin, and geraniin, which was consistent with previous reports [9]. The extract of the *N. lappaceum* seed was found to be dominated by flavonoids based on molecular networking. In summary, the extracts of the *N. lappaceum* fruit peel, pulp and seed were dominated by sugars, tannins (ellagitannin and gallotannin) and flavonoids, respectively, from visualized molecular networking.

### 3.2. Bioactivity-Guided Isolation and Dereplication of the Active Compounds

**Figure 1:**
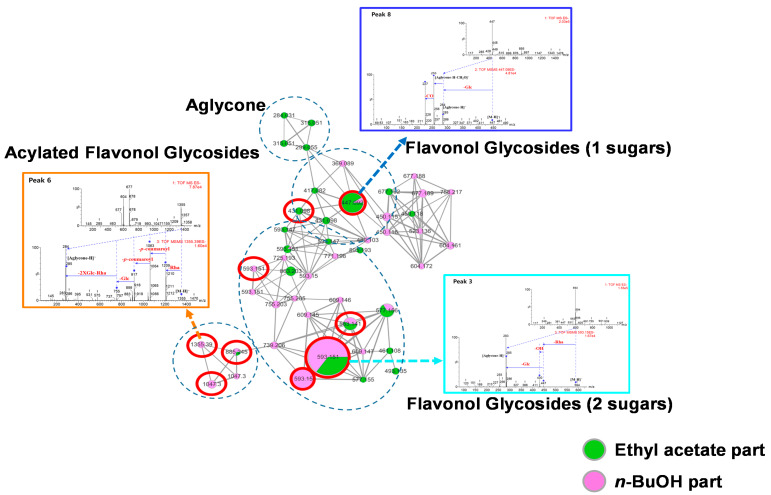
Bioactive molecular networking of the ethyl acetate (green cluster) and *n*-BuOH (pink cluster) soluble fractions of the *N. lappaceum* seed extract. Detailed conditions for molecular networking were provided in the Supplementary Materials (Figure S41).

The p16INK4A and SA-β-gal luciferase reporter gene analyses of the four fractions obtained from the seed extracts were performed continuously to support further bioactivity-guided isolation (Figures S45 and S46 in Supplementary Materials). Among the four fractions, the EA and *n*-BuOH fractions were selected as the biologically active fractions and then analyzed using HPLC-qTOF-MS/MS to predict the biologically active compounds. The molecular networking of the biologically active fractions suggested that the main active compounds in the EA and *n*-BuOH fractions were flavonol glycosides and acylated flavonol glycosides according to their characteristic MS/MS fragments (Figure 1).

**Figure 2:**
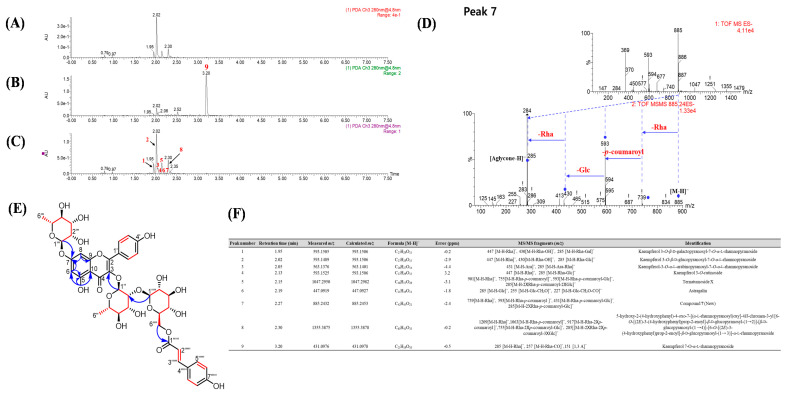
DAD (UV) chromatograms of (**A**) the crude extract, (**B**) the ethyl acetate (EA)-soluble fraction, and (**C**) the *n*-BuOH-soluble fraction of *N. lappaceum* seeds at 280 nm. Nine compounds (**1**–**9**) were quantitatively analyzed by UHPLC/ESI-qTOF-MS/MS in negative ion mode. The nine major compounds were isolated and their structures were determined by various spectroscopic techniques. (**D**) The fragmentation routes of compound **7** in *N. lappaceum* seeds. (**E**) Key HMBC (blue arrows) and the ^1^H-^1^H COSY (red) correlations of compound **7**. (**F**) Peak assignment of the nine flavonol glycosides of *N. lappaceum* seeds.

The structures of the nine major compounds in the EA and *n*-BuOH fractions were predicted based on their MS/MS fragment patterns (Figure 2A–C and F). Peak 1 showed a product ion at *m*/*z* 285 corresponding to the loss of a rhamnose (146 Da) and a galactose (162 Da) from the parent ion peak, and this was the characteristic *m*/*z* of kaempferol. Peak 2 was the most abundant and indicated structural differences due to the presence of a glucose instead of a galactose. Peak 3 generated product ions at *m*/*z* 431 and 285 with sequential losses of 132 Da (Ara) and 146 Da (Rha), and peak 4 showed a product ion at *m*/*z* 285, corresponding to the loss of a rhamnose (146 Da) and a glucose (162 Da) from the parent ion peak. Peak 5 presented product ions at *m*/*z* 901, 755, 593 and 285 from the losses of 146 Da (Rha), 146 Da (p-coumaroyl), 162 Da (Glc) and 146 Da (Rha) with 162 Da (Glc), respectively. Peak 6 showed product ions at *m*/*z* 285, 255 and 227, corresponding to the loss of 162 Da (Glc). The precursor ion of peak 8 was *m*/*z* 1355.3 [M − H]^─^, and the fragment ions were produced by the loss of 146 Da (Rha), 292 Da (2 × p-coumaroyl), 162 Da (Glc) and 146 Da (Rha) with two glucose moieties (324 Da). Peak 9 presented fragments at *m*/*z* 285, 257 and 151 from sequential losses of rhamnose (146 Da), suggesting the presence of a kaempferol group. Specifically, peak 7 showed different fragmentation patterns (Figure 2D) with sequential losses of 146 Da (Rha), 146 Da (p-coumaroyl), 162 Da (Glc) and 146 Da (Rha), suggesting that it was a new compound. Thus, six known flavonol glycosides as well as one new and two known acylated flavonol glycosides were predicted and isolated for structural confirmation. Finally, these compounds were determined to be kaempferol 3-O-β-d-galactopyranosyl-7-O-α–l-rhamnopyranoside (**1**) [13], kaempferol 3-O-β-d-glucopyranosyl-7-O-α–l-rhamnopyranoside (**2**) [14], kaempferol 3-O-α-l-arabinopyranosyl-7-O-α–l-rhamnopyranoside (**3**) [15], kaempferol 3-O-rutinoside (**4**) [16], ternatumoside X (**5**) [17], astragalin (**6**) [18], 5-hydroxy-2-(4-hydroxyphenyl)-4-oxo-7-[(α-l-rhamnopyranosyl)oxy]-4H-chromen-3-yl [6-O-[(2E)-3-(4-hydroxyphenyl)prop-2-enoyl]-β-d-glucopyranosyl-(1→2)]-[β-d-glucopyranosyl-(1→4)]-[6-O-[(2E)-3-(4-hydroxyphenyl)prop-2-enoyl]-β-d-glucopyranosyl-(1→3)]-α-l-rhamnopyranoside (**8**) [19,20], and kaempferol 7-O-α-l-rhamnopyranoside (**9**) [21] by comparing their physicochemical properties and NMR spectra with those previously reported.

### 3.3. Isolation and Identification of New Compound ***7***

Compound **7** was isolated as a pale-yellow, gummy solid with ${\left[\alpha \right]}_{D}^{25}$ −83.3 (*c* 0.1, MeOH). The HRESIMS spectrum of compound **7** revealed a negative ion peak [M − H]^─^ at *m*/*z* 885.2432 (calcd for C_42_H_45_O_21_, 885.2453) and was assigned the formula C_42_H_46_O_21_. The aglycone of **7** was suggested to be acylated kaempferol glycoside, corresponding to the MS^2^ product ions such as 739 (−146 Da, −rhamnopyranoyl), 593 (−146 Da, −p-coumaroyl), 431 (−162 Da, −glucopyranoyl) and 285 (−146, −rhamnopyranoyl) (Figure 2D). The aromatic regions of the ^1^H and ^13^C NMR spectra (Table S3 in Supplementary Materials) showed two AA′XX′ systems [*δ*_H_ 7.75 (2H, d, *J* = 8.7 Hz), *δ*_C_ 130.6; 7.35 (2H, d, *J* = 8.6 Hz), 130.0; 6.92 (2H, d, *J* = 8.7 Hz), 115.4; and 6.68 (2H, d, *J* = 8.6 Hz), 115.6], an AX system [*δ*_H_ 6.65 (1H, br s), *δ*_C_ 94.5 and 6.40 (1H, br s), 99.4] and two pairs of doublets with a large coupling constant [*δ*_H_ 6.14 (1H, d, *J* = 15.9 Hz), *δ*_C_ 113.8 and 6.41 (1H, d, *J* = 15.9 Hz), 144.9], which are typical signals for an acylated kaempferol structure. Moreover, two α-rhamnopyranoyl moieties [(*δ*_H_ 5.63 (1H, s), *δ*_C_ 100.6 and *δ*_H_ 5.51 (1H, s), 98.5)] and one β-glucopyranoyl moiety [*δ*_H_ 4.32 (1H, d, *J* = 7.9 Hz), *δ*_C_ 106.0] were indicated by these spectra along with the positions of those sugars and the p-coumaroyl moiety. In the HMBC spectrum (Figure S28), the correlations between H-1″ and C-2 (*δ*_C_ 134.6) and between H-1‴ and C-7 (*δ*_C_ 161.7) showed the connectivity of the sugars linked to the aglycone. The downfield shielding of C-2″ (*δ*_C_ 81.8) indicated a β-glucose attached to C-2″, and this connection was confirmed by the HMBC cross peak between H-1⁗ and C-2″. Additionally, the connection of a p-coumaroyl moiety to C-6⁗ of the glucose was confirmed by the HMBC cross peak from H-6⁗ to C-1′′′′′ (*δ*_C_ 166.3). Therefore, the structure of compound **7** was completely determined and is shown in Figure 2E.

### 3.4. Effect of the Fractions and Compounds **1**–**9** at the Transcriptional Level of p16INK4A and Senescence-Associated-β-Galactosidase

**Figure 3:**
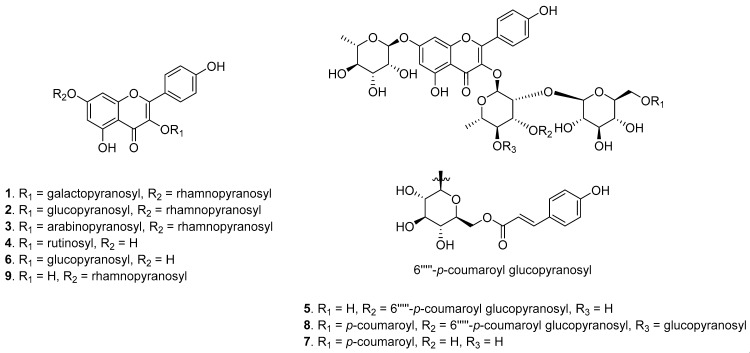
Chemical structures of compounds **1**–**9**.

**Figure 4:**
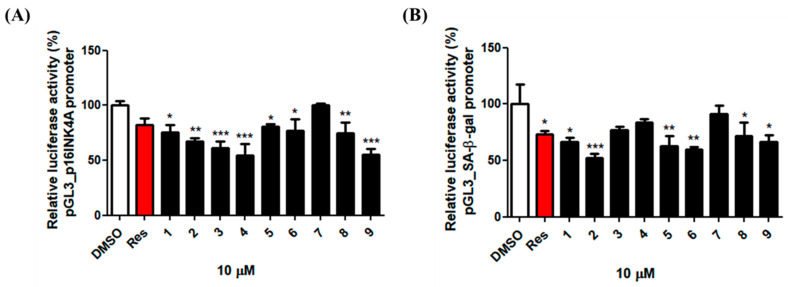
Effects of compounds **1**–**9** (10 μM) on the p16INK4A and GLB1 transcription in the human dermal fibroblasts. Human dermal fibroblasts were transiently co-transfected with the pGL3-p16INK4A (**A**) or pGL3-GLB1 (**B**) promoter with β-galactosidase as a transfection control. Cells were treated overnight with the compounds **1**–**9** (10 μM). The luciferase activity was determined as the ratio of the firefly/Renilla luciferase activities. The activities of the p16Ink4A promoter and the GLB1 promoter in the presence of the compounds **1**–**9** relative to that in the absence of the compounds **1**–**9** are shown. * *p* < 0.05, ** *p* < 0.01, and *** *p* < 0.001, compared with control.

To assess the cytotoxicities of the nine isolated compounds (Figure 3) toward the HDFs, the 10 μM single compounds were treated with the cells to confirm that the nine compounds were not toxic to the cell (Figure S46 in Supplementary Materials). As elevated SA-β-gal activity and p16INK4A are both known biomarkers of cellular senescence and skin aging, we investigated the effects of the isolated compounds on the promoter activities of these two unique senescence markers. As a result of compound treatment at a concentration of 10 μM, some compounds significantly reduced luciferase activity driven by the p16INK4A and SA-β-gal promoters (Figure 4). Resveratrol was used as a positive control to determine the down regulatory effects of p16INK4A and SA-β–gal promoter activity [22]. These data demonstrated that the isolated compounds were present in the EA and *n*-BuOH fractions and generally inhibited the expression of the p16INK4A and SA-β–gal genes at the transcriptional level, which are known biomarkers of cellular senescence and skin aging.

### 3.5. Senomorphic Effects of Compounds ***2***, ***4*** and ***9*** in Rescuing Replicative Senescence and Inhibiting SASP

**Figure 5:**
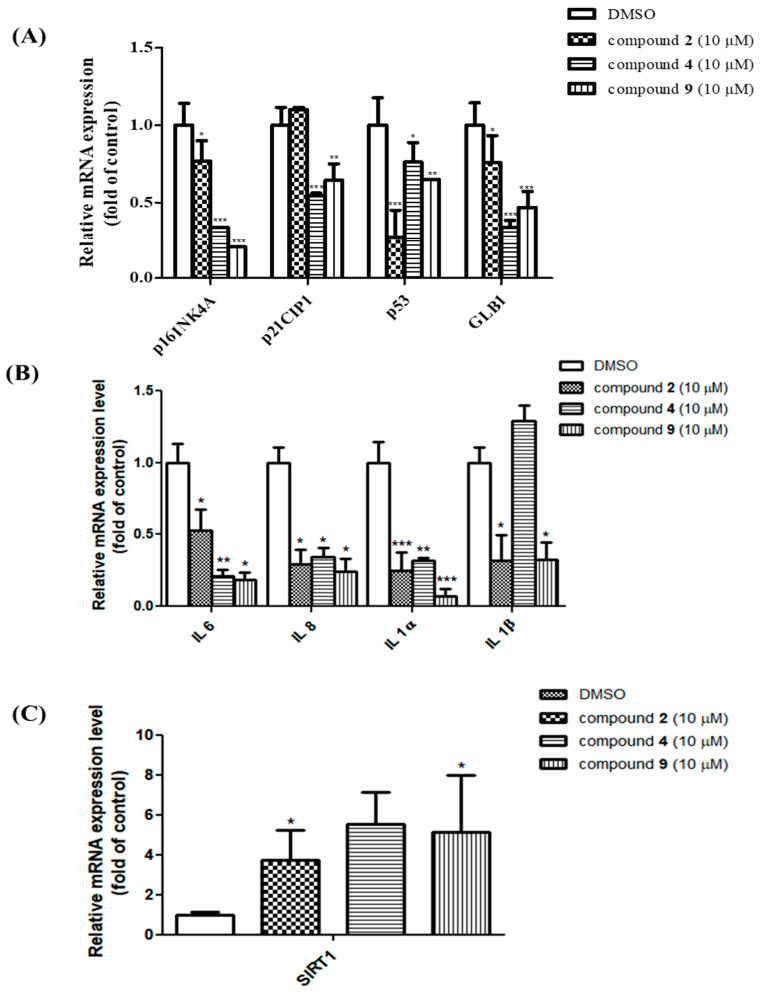
Effects of the isolated compounds on the cell cycle arrest genes, the senescence-associated secretory phenotype (SASP) mRNA expression and the sirtuin 1 (SIRT1) in the senescent human dermal fibroblasts (HDFs). RNA was isolated, and the relative mRNA levels of p16INK4A, p53, p21, SA-β-gal (**A**), SASP (**B**) and SIRT1 (**C**) between the treatment with DMSO and with the 10 μM compounds **2**, **4** and **9** were determined by qRT-PCR. For data normalization, 18S rRNA was used as an internal control. * *p* < 0.05, ** *p* < 0.01, and *** *p* < 0.001, compared with control.

During senescence, cells secrete various molecules called senescence-associated secretory phenotypes (SASPs), including proinflammatory cytokines, chemokines and proteases into their surrounding environment. In a previous report, the effects of flavonoids (apigenin, quercetin, kaempferol and naringenin) on SASP from bleomycin-induced senescence in BJ fibroblasts were investigated [7]. Kaempferol glycosides were also isolated from *N. lappaceum,* suggesting that the elevated levels of SASPs in aged HDFs could be reduced. Thus, HDFs were treated with 10 μM test compounds for 72 h to determine their roles in senescent states. A previous report investigated the effects of flavonoids (apigenin, quercetin, kaempferol and naringenin) on SASP via the suppression of the NF-*κ*B pathway from the bleomycin-induced senescence in BJ fibroblasts [8]. The treatment with compounds **2**, **4** and **9** showed a decrease in the mRNA levels of most aging-related genes (Figure 5A) and SASP, i.e., IL-6, IL-8, IL-1α, and IL-1β, except for p21 of **2** and an IL-1β of **9** (Figure 5B). SIRT1 was shown to prevent aging by blocking senescence markers such as p16INK4A, p53, p21, SA-β-gal and SASPs. To investigate the potency of these three compounds as SIRT1 activators, mRNA expression levels were measured through real-time PCR (Figure 5C). In particular, compounds **2**, **4** and **9** were found to significantly activate the expression of SIRT1, with at least a three- to five-fold increase in senescent HDFs after 72 h of treatment with the final hit compounds. These results suggest that compounds **2**, **4** and **9** might be new candidate senomorphics that function by suppressing the expression of age-associated genes as well as activating SIRT1.

### 3.6. Compounds ***2***, ***4*** and ***9*** Attenuate the Senescence Phenotype

**Figure 6:**
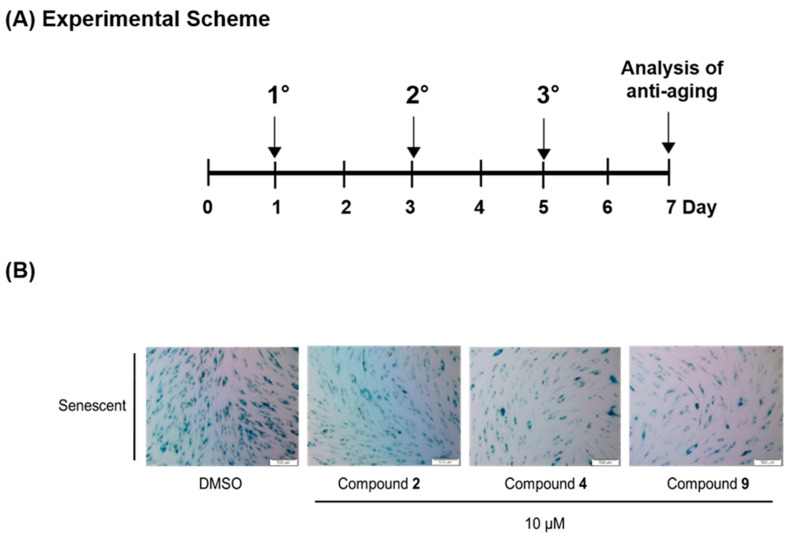
Experimental scheme and the result of the senescence-associated β-galactosidase staining. Senescent HDFs were treated with a vehicle or 10 μM compounds **2**, **4** or **9** three times over 6 days (**A**). Using an SA-β-galactosidase staining kit (at# 9860; Cell Signaling Technology, Denver, MA, USA), the SA-β-galactosidase-positive cells were stained according to the manufacturer’s instructions. Cells stained for SA-β-gal activity were observed and bright field images were taken with a fluorescence microscope (Olympus ix70, Olympus Corporation, Tokyo, Japan) (**B**). In addition, the quantification of the β-galactosidase staining was provided in the Supplementary Materials (Figure S48).

Recently, the identification of senescent cells by colorimetric assays, especially SA-β-gal staining, has become feasible. The activity of SA-β-gal, a lysosomal enzyme, is elevated in senescent cells and it is particularly detectable at pH 6 [23]. To confirm that our compounds can prevent the process of cellular senescence, the SA-β-gal activity was assessed in all the senescent HDF groups. Staining was observed in 90% of the senescent HDFs, whereas young HDFs showed little or no staining (Figure S47 in Supplementary Materials). The senescent HDFs were treated with 10 μM compounds **2**, **4** and **9** three times over six days, as shown in the experimental scheme (Figure 6A). The results show that the relative ratio of the stained cells between the treated and untreated (control) groups was significant, as observed using a microscope (Figure 6B). Therefore, compounds **2**, **4** and **9** were effective in attenuating the senescence phenotype induced by replicative senescence.

## 4. Discussion

In previous reports, substantial capacities to suppress SASPs [8] and antioxidant effects were attributed to flavonoids such as kaempferol and apigenin. Based on molecular networking and senescence-associated p16INK4A and SA-β-gal promoter activity assays, we found that the *N. lappaceum* seed extract had stronger activity than the extracts of its pulp (sugar), peel (ellagitannin and gallotannin) and total extracts. From the DAD chromatograms and MS/MS fragmentation profile-based molecular networks, it was determined that the *N. lappaceum* seeds contained flavonoids, while the pulp and peel primarily contained sugar and tannins, respectively. The EA and *n*-BuOH fractions showed higher senomorphic activities than the *n*-hexane and water fractions from the *N. lappaceum* seed extract based on senescence-related biomarker luciferase promoter activity. The bioactivity-guided fractionation and MS/MS profiling via molecular networking suggested that the flavonol glycosides and acylated flavonol glycosides that were present were the active constituents in the EA and *n*-BuOH fractions. In the present study, one new compound (**7**) along with eight known compounds (**1**–**6**, **8** and **9**) were isolated from the seeds of *N. lappaceum.* This study demonstrated new senomorphic candidates to prevent replicative senescence in HDFs. The main finding is that compounds **2**, **4** and **9** not only decrease the expression of cell cycle arrest genes such as p16INK4A and p53, but also increase SIRT1 activity. However, p21, as an effector of senescence, was inhibited only by compounds **4** and **9**. The p21 as an effector of senescence was controlled by the p53 and TGFβ–SMAD axis. Compounds **4** and **9** may inhibit both p53−p21 and TGFβ signaling, thus p21 showed significant down-regulation. However, compound **2** may only inhibit the p53−p21 pathway, which may result in p53 down-regulation without p21 inhibition (Figure S49 in Supplementary Materials). Furthermore, compounds **2**, **4** and **9** suppressed SASPs such as IL-6, IL-8, IL-1α and IL-1β and minimized SA-β-gal-positive senescent HDFs. This reveals the potential effects of compounds **2**, **4** and **9** from the seeds of *N. lappaceum* in ameliorating age-related diseases, improving health and extending the human lifespan.

## 5. Conclusions

In this study, extracts and fractions obtained from *N. lappaceum* seeds showed better senomorphic activity than the other parts (peels and pulps), and senomorphic activity was also observed in isolated compounds **2**, **4**, and **9**. Food-derived senomorphic molecules are relatively less toxic and are likely to be developed as candidates for the treatment of aging-related diseases. *N. lappaceum* seeds found industrial value by discovering potential senomorphic candidates through this study. Therefore, extracts, fractions and compounds **2**, **4**, and **9** obtained from the seeds of *N. lappaceum* could suggest the possibility for use as cosmetics and therapeutics for aging-related diseases in the future.

## Supplementary Materials

The following are available online at https://www.mdpi.com/2072-6643/12/5/1430/s1, Figure S1:^1^H NMR spectrum of compound **1** (600 MHz, DMSO-*d*_6_), Figure S2:^13^C NMR spectrum of compound **1** (150 MHz, DMSO-*d*_6_), Figure S3: IR spectrum of compound **1**, Figure S4: Fragmentation pathway of compound **1**, Figure S5: ^1^H NMR spectrum of compound **2** (500 MHz, DMSO-*d*_6_), Figure S6:^13^C NMR spectrum of compound **2** (125 MHz, DMSO-*d*_6_), Figure S7: IR spectrum of compound **2**, Figure S8: Fragmentation pathway of compound **2**, Figure S9:^1^H NMR spectrum of compound **3** (600 MHz, DMSO-*d*_6_), Figure S10:^13^C NMR spectrum of compound **3** (150 MHz, DMSO-*d*_6_), Figure S11: IR spectrum of compound **3**, Figure S12: Fragmentation pathway of compound **3**, Figure S13:^1^H NMR spectrum of compound **4** (400 MHz, Methanol-*d*_4_), Figure S14:^13^C NMR spectrum of compound **4** (100 MHz, Methanol-*d*_4_), Figure S15: IR spectrum of compound **4**, Figure S16: Fragmentation pathway of compound **4**, Figure S17:^1^H NMR spectrum of compound **5** (500 MHz, Methanol-*d*_4_), Figure S18:^13^C NMR spectrum of compound **5** (125 MHz, Methanol-*d*_4_), Figure S19: IR spectrum of compound **5**, Figure S20: Fragmentation pathway of compound **5**, Figure S21:^1^H NMR spectrum of compound **6** (600 MHz, DMSO-*d*_6_), Figure S22:^13^C NMR spectrum of compound **6** (150 MHz, DMSO-*d*_6_), Figure S23: IR spectrum of compound **6**, Figure S24: Fragmentation pathway of compound **6**, Figure S25:^1^H NMR spectrum of compound **7** (600 MHz, DMSO-*d*_6_), Figure S26:^13^C NMR spectrum of compound **7** (150 MHz, DMSO-*d*_6_), Figure S27: HSQC NMR spectrum of compound **7** (600 MHz, DMSO-*d*_6_), Figure S28: HMBC NMR spectrum of compound **7** (600 MHz, DMSO-*d*_6_), Figure S29: ^1^H-^1^H COSY NMR spectrum of compound **7** (600 MHz, DMSO-*d*_6_), Figure S30: IR spectrum of compound **7**, Figure S31: HRESIMS spectrum of compound **7**, Figure S32:^1^H NMR spectrum of compound **8** (400 MHz, Methanol-*d*_4_), Figure S33:^13^C NMR spectrum of compound **8** (100 MHz, Methanol-*d*_4_), Figure S34: IR spectrum of compound **8**, Figure S35: Fragmentation pathway of compound **8**, Figure S36:^1^H NMR spectrum of compound **9** (500 MHz, DMSO-*d*_6_), Figure S37:^13^C NMR spectrum of compound **9** (125 MHz, DMSO-*d*_6_), Figure S38: IR spectrum of compound **9**, Figure S39: Fragmentation pathway of compound **9**, Figure S40: Sugar Analysis, Figure S41: Molecular networking, Figure S42: Effect of *N. lappaceum* peel, pulp and seed on p16Ink4A and SA-β-gal promoter activity in human dermal fibroblasts, Figure S43: Annotation of the molecular networking of the crude extract from *N. lappaceum* pulp (red nodes), peel (aquamarine nodes), seed (green nodes) which shows flavonoid, ellagitannin, gallotannin and sugar, Figure S44: DAD chromatograms of crude extract of *N. lappaceum* pulp (A), peel (B) and seed (C) at 280 nm. (D) major compounds of pulp, peel and seed extracts of *N. lappaceum* identified in corresponding MS/MS fragmentation profiles, Figure S45: Effect of crude extract and four fractions from *N. lappaceum* seeds on p16INK4A and SA-β-gal transcription in human dermal fibroblasts, Figure S46: The cytotoxicity effect of total extract and four fractions 20 μg/mL (A) and 10 μM compounds **1**–**9** obtained from *N. lappaceum* seeds (B) both in young and senescent HDFs, Figure S47: Senescence-associated β-galactosidase staining of HDFs. Young and aged HDFs stained for 24h for SA-β-galactosidase. Figure S48: Quantification of senescence-associated β-galactosidase staining, Figure S49: Mechanism of action of compounds **2**, **4** and **9** isolated from rambutan, Table S1: ^1^H NMR data for compounds **1**–**6**, **8** and **9**, Table S2: ^13^C NMR data for compounds **1**–**6**, **8** and **9**, Table S3. ^1^H and ^13^C NMR data for compound **7**, Table S4: Sequence of primer used in qRT-PCR.
