# Supplementary material for: Metabolite Profiling of Rambutan (Nephelium lappaceum L.) Seeds Using UPLC-qTOF-MS/MS and Senomorphic Effects in Aged Human Dermal Fibroblasts
Source: Nutrients. 2020 May 15;12(5):1430. doi: 10.3390/nu12051430 (PMC7284571; doi:10.3390/nu12051430)
Supplement: Supplementary file 1 [file nutrients-12-01430-s001.pdf]

Metabolite Profiling of Rambutan (*Nephelium lappaceum* L.) Seeds using UPLC-qTOF-MS/MS and Senomorphic Effects in Aged Human Dermal Fibroblasts

Yae Rin Lee [1,†], Hyo Moon Cho [1,†], Eun Jin Park [1], Mi Zhang [1], Thi Phuong Doan [1], Ba Wool Lee [1], Kyung A Cho [2] and Won Keun Oh [1,*]

[1] Korea Bioactive Natural Material Bank, Research Institute of Pharmaceutical Sciences, College of Pharmacy, Seoul National University, Seoul 08826, Republic of Korea; leeyaerin93@snu.ac.kr (Y.R.L); chgyans@naver.com (H.M.C); eunjin_p@snu.ac.kr (E.J.P); mintazhang@snu.ac.kr (M.Z); phuongdoan@snu.ac.kr (T.P.D); paul36@snu.ac.kr (B.W.L); wkoh1@snu.ac.kr (W.K.O)

[2] Department of Biochemistry, Chonnam National University Medical School, Gwangju 51828, Korea; kacho@jnu.ac.kr (K.A.C)

* Correspondence: wkoh1@snu.ac.kr; Tel.: +82-2-880-7872

† These authors contributed equally to this work.

Contents:

Figure S1: ^{1}H NMR spectrum of compound **1** (600 MHz, DMSO-d_6).

Figure S2: ^{13}C NMR spectrum of compound **1** (150 MHz, DMSO-d_6).

Figure S3: IR spectrum of compound **1.**

Figure S4: Fragmentation pathway of compound **1.**

Figure S5: ^{1}H NMR spectrum of compound **2** (500 MHz, DMSO-d_6).

Figure S6: ^{13}C NMR spectrum of compound **2** (125 MHz, DMSO-d_6).

Figure S7: IR spectrum of compound **2.**

Figure S8: Fragmentation pathway of compound **2.**

Figure S9: ^{1}H NMR spectrum of compound **3** (600 MHz, DMSO-d_6).

Figure S10: ^{13}C NMR spectrum of compound **3** (150 MHz, DMSO-d_6).

Figure S11: IR spectrum of compound **3.**

Figure S12: Fragmentation pathway of compound **3.**

Figure S13: ^{1}H NMR spectrum of compound **4** (400 MHz, Methanol-d_4).

Figure S14: ^{13}C NMR spectrum of compound **4** (100 MHz, Methanol-d_4).

Figure S15: IR spectrum of compound **4.**

Figure S16: Fragmentation pathway of compound **4.**

Figure S17: ^{1}H NMR spectrum of compound **5** (500 MHz, Methanol-d_4).

Figure S18: ^{13}C NMR spectrum of compound **5** (125 MHz, Methanol-d_4).

Figure S19: IR spectrum of compound **5.**

Figure S20: Fragmentation pathway of compound **5.**

Figure S21: ^{1}H NMR spectrum of compound **6** (600 MHz, DMSO-d_6).

Figure S22: ^{13}C NMR spectrum of compound **6** (150 MHz, DMSO-d_6**).**

Figure S23: IR spectrum of compound **6.**

Figure S24: Fragmentation pathway of compound **6.**

Figure S25: ^{1}H NMR spectrum of compound **7** (600 MHz, DMSO-d_6).

Figure S26: ^{13}C NMR spectrum of compound **7** (150 MHz, DMSO-d_6).

Figure S27: HSQC NMR spectrum of compound **7** (600 MHz, DMSO-d_6).

Figure S28: HMBC NMR spectrum of compound **7** (600 MHz, DMSO-d_6).

Figure S29: ^1H-^1H COSY NMR spectrum of compound **7** (600 MHz, DMSO-d_6).

Figure S30: IR spectrum of compound **7.**

Figure S31: HRESIMS spectrum of compound **7.**

Figure S32:^1H NMR spectrum of compound **8** (400 MHz, Methanol-d_4).

Figure S33:^{13}C NMR spectrum of compound **8** (100 MHz, Methanol-d_4).

Figure S34: IR spectrum of compound **8.**

Figure S35: Fragmentation pathway of compound **8.**

Figure S36:^1H NMR spectrum of compound **9** (500 MHz, DMSO-d_6).

Figure S37:^{13}C NMR spectrum of compound **9** (125 MHz, DMSO-d_6).

Figure S38: IR spectrum of compound **9.**

Figure S39: Fragmentation pathway of compound **9.**

Figure S40: Sugar Analysis. Compound **7** (2.5 mg) was heated in 0.1 mL at 110°C for 1.5 h of 2 M HCl. After acid hydrolyzed sample was neutralized with Na_2CO_3 and dried. The residue was derivatized with L-cysteine methyl ester hydrochloride in anhydrous pyridine (200 μL, 60 °C, 1 h) and subsequently added phenylisothiocyanate (1 μL, 60 °C, 1 h). The

sugar derivatives from compound **7** were compared with standard sugar derivatives by the HPLC analysis and were confirm the absolute configuration of these sugars to be D-glucose, and L-rhamnose.

Figure S41: Molecular Networking

Figure S42: Effect of *N. lappaceum* peel, pulp and seed on p16Ink4A and SA-β-gal promoter activity in human dermal fibroblasts.

Figure S43: Annotation of the molecular networking of the crude extract from *N. lappaceum* pulp (red nodes), peel (Aquamarine nodes), seed (green nodes) which shows flavonoid, ellagitannin, gallotannin and sugar.

Figure S44: DAD chromatograms of crude extract of *N. lappaceum* pulp (A), peel (B), and seed (C) at 280 nm. (D) major compounds of pulp, peel and seed extracts of *N. lappaceum* identified in corresponding MS / MS fragmentation profiles.

Figure S45: Effect of total extract and four fractions on p16INK4A and SA-β-gal transcription in human dermal fibroblasts. Human dermal fibroblasts were transiently co-transfected with pGL3-p16ink4a (A) or pGL3-glb1 (B) promoter with β-galactosidase as a transfection control.

Figure S46: The cytotoxicity effect of total extract and four fractions 20 μg/mL (A) and 10 μM compounds **1-9** obtained from *N. lappaceum* seeds (B) both in young and senescent

HDFs. After 24 h of incubation with tested fractions and compounds, the MTT assay was carried out as described in the experimental section.

Figure S47: Senescence-associated β-galactosidase staining of HDFs. Young and aged HDFs stained for 24h for SA-β-galactosidase. Images of young HDFs (passage 14) had little staining, while aged HDFs (passage 39) stained blue.

Figure S48: Quantification of senescence-associated β-galactosidase staining. Aged HDF cells were treated three times over 6 days with vehicle or 10 μM compounds **2**, **4**, and **9** and quantified through images obtained from a fluorescence microscope.

Figure S49: Mechanism of action of compounds **2**, **4**, and **9** isolated from Rambutan.

Table S1: ^{1}H NMR data for compounds **1**–**6, 8** and **9**.

Table S2: ^{13}C NMR data for compounds **1**–**6, 8** and **9**.

Table S3. ^{1}H and ^{13}C NMR data for compound **7.**

Table S4: Sequence of primer used in qRT-PCR.

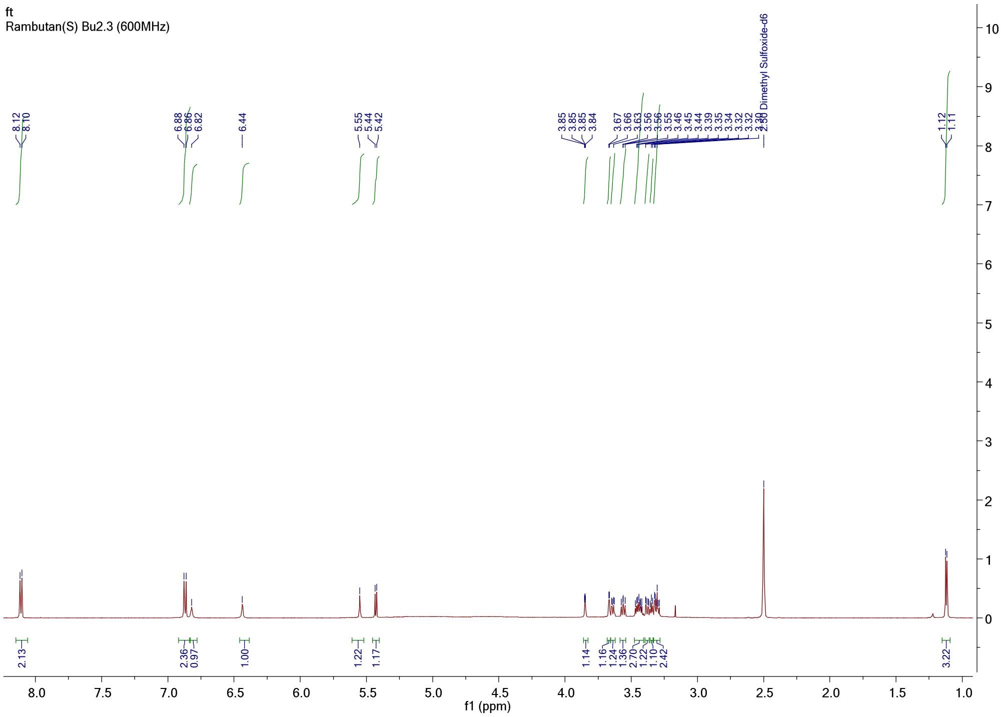

Figure S1:^{1}H NMR spectrum of compound **1** (600 MHz, DMSO-d_6).

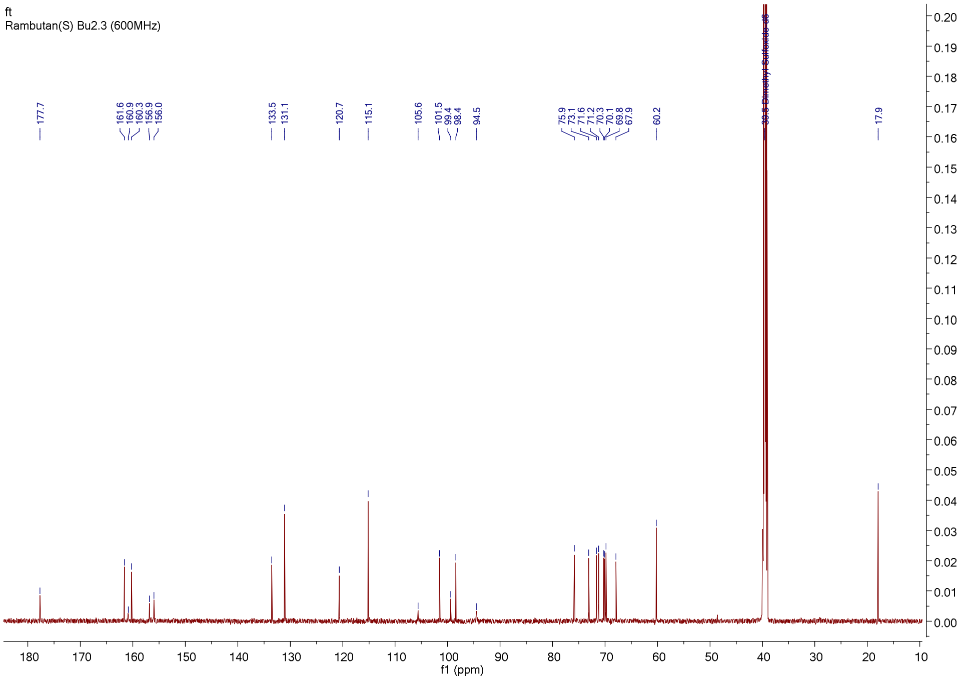

Figure S2:^{13}C NMR spectrum of compound **1** (150 MHz, DMSO-d_6).

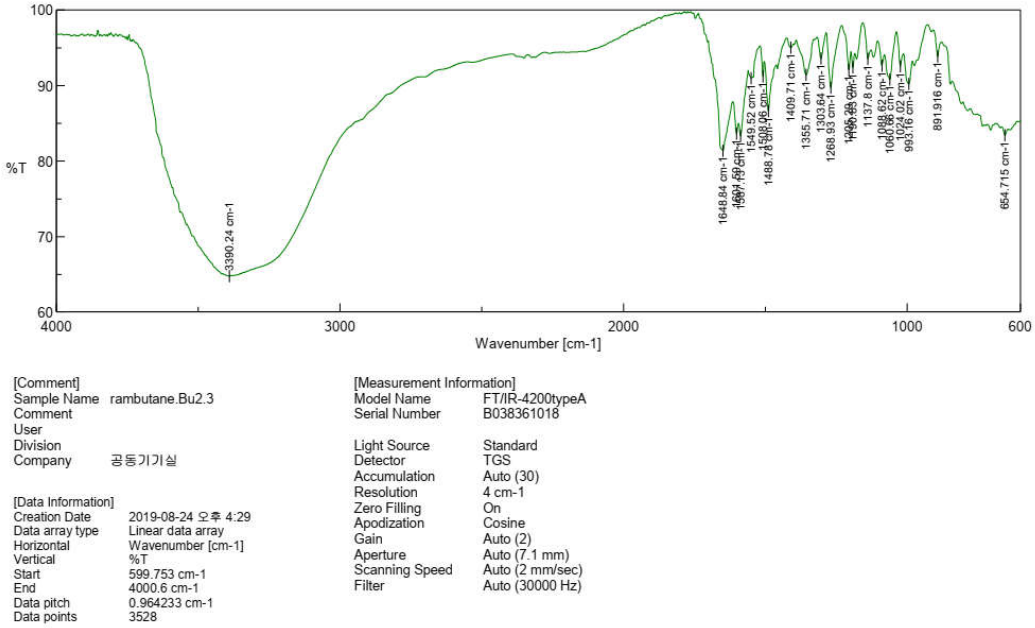

Figure S3: IR spectrum of compound **1.**

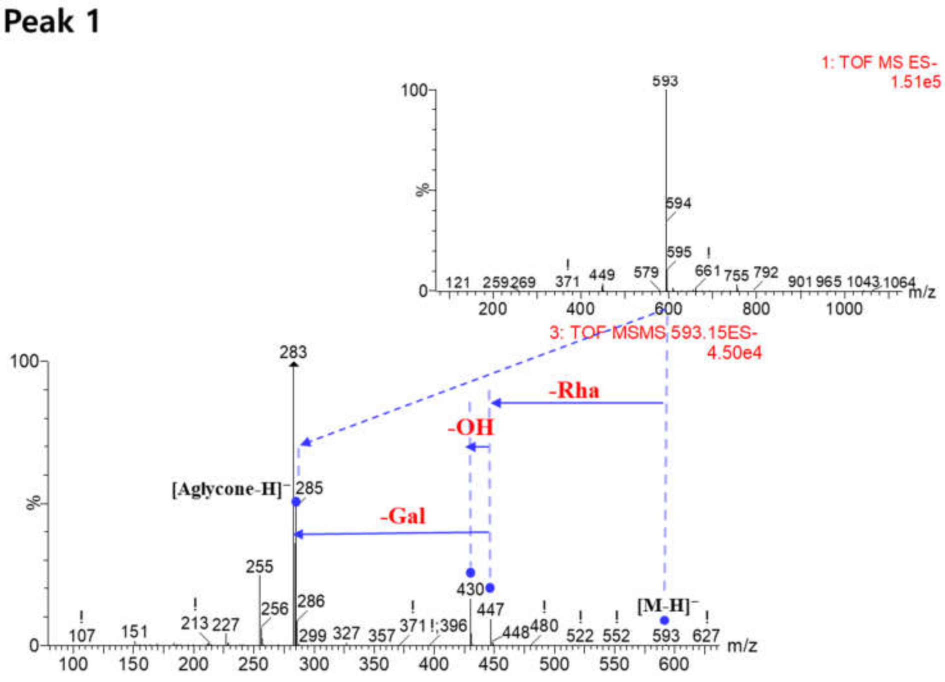

Figure S4: Fragmentation pathway of compound **1.**

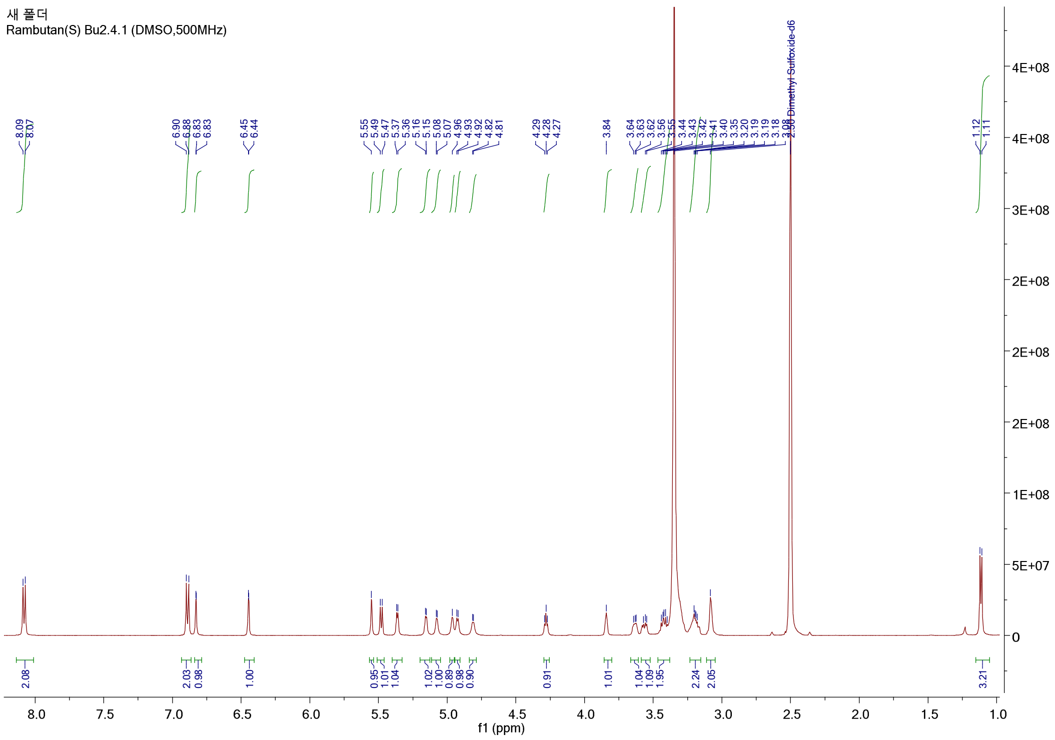

Figure S5: ^{1}H NMR spectrum of compound **2** (500 MHz, DMSO-d_6).

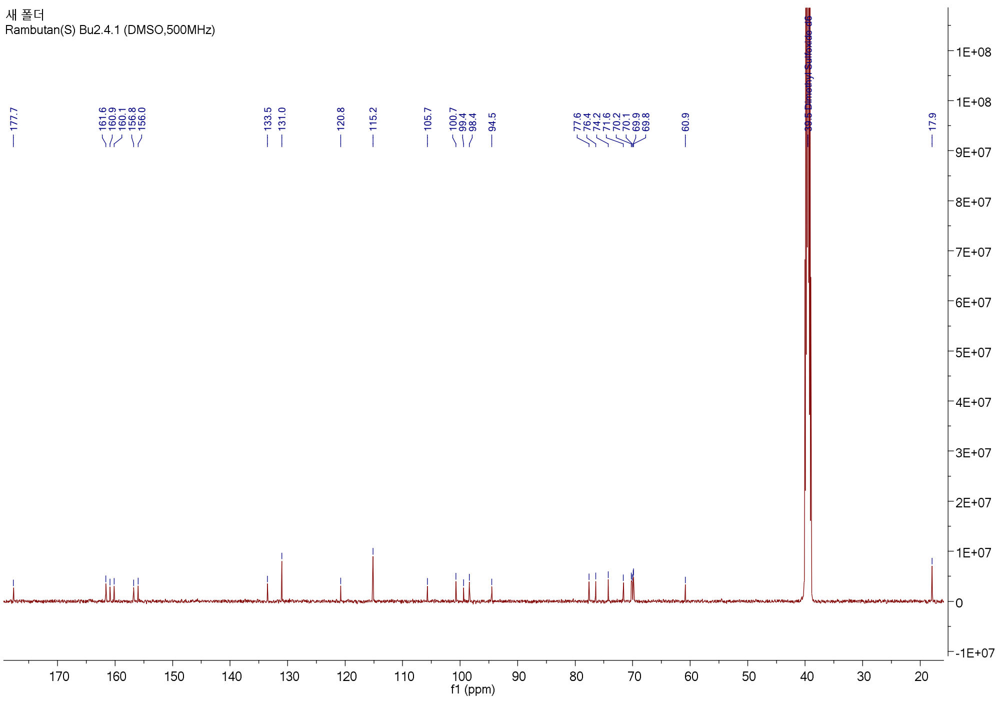

Figure S6: ^{13}C NMR spectrum of compound **2** (125 MHz, DMSO-d_6).

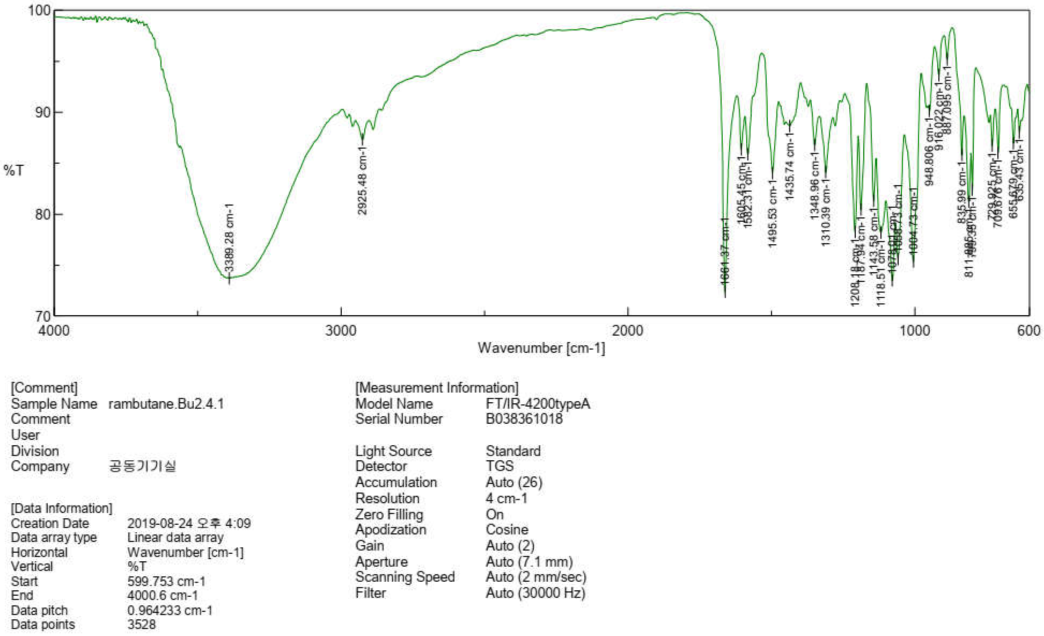

Figure S7: IR spectrum of compound **2.**

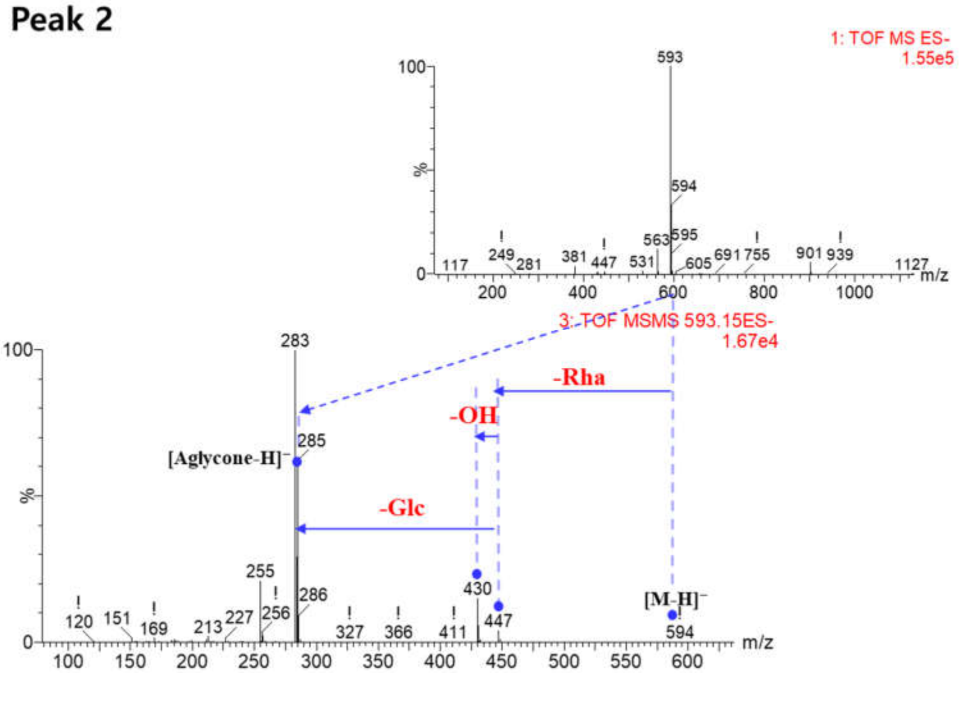

Figure S8: Fragmentation pathway of compound **2.**

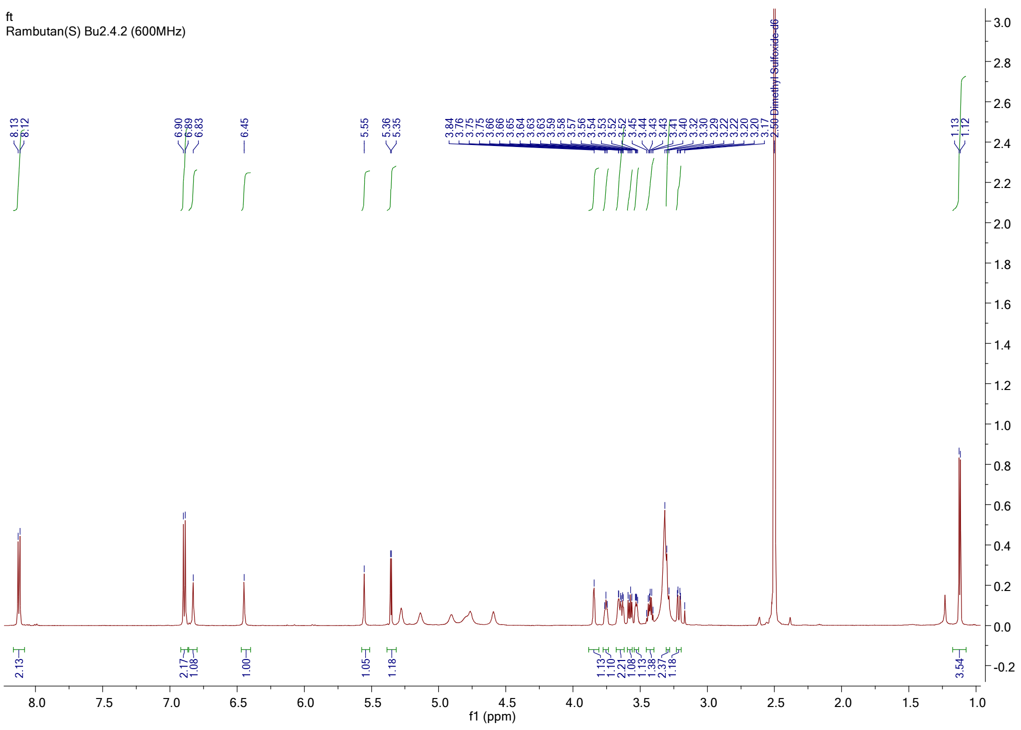

Figure S9: ^{1}H NMR spectrum of compound **3** (600 MHz, DMSO-d_6).

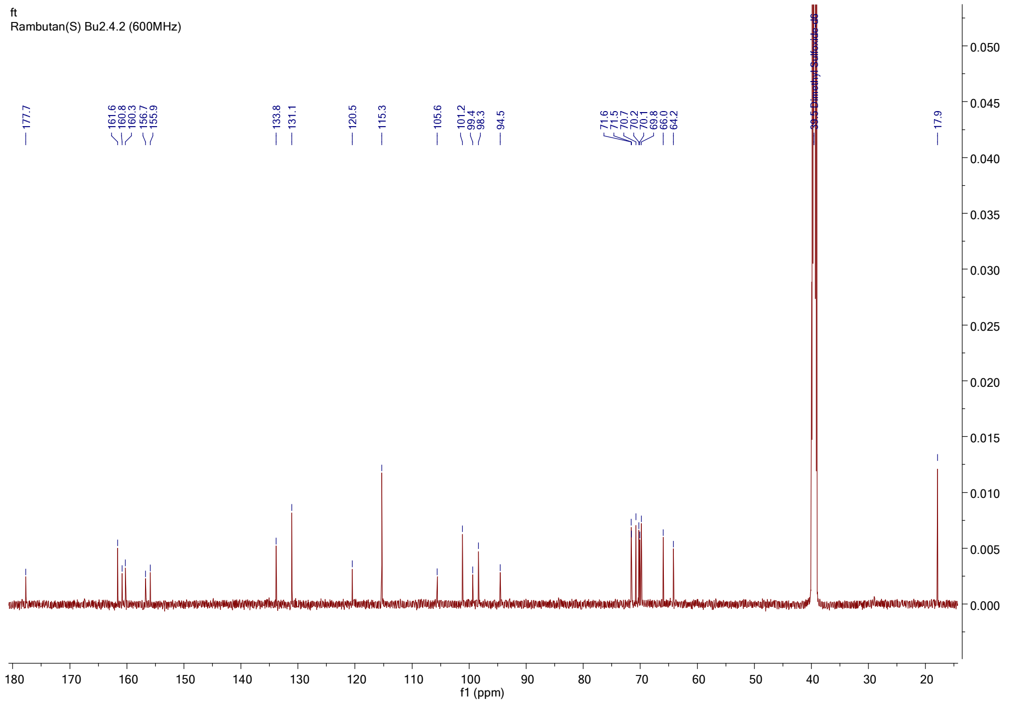

Figure S10: ^{13}C NMR spectrum of compound **3** (150 MHz, DMSO-d_6).

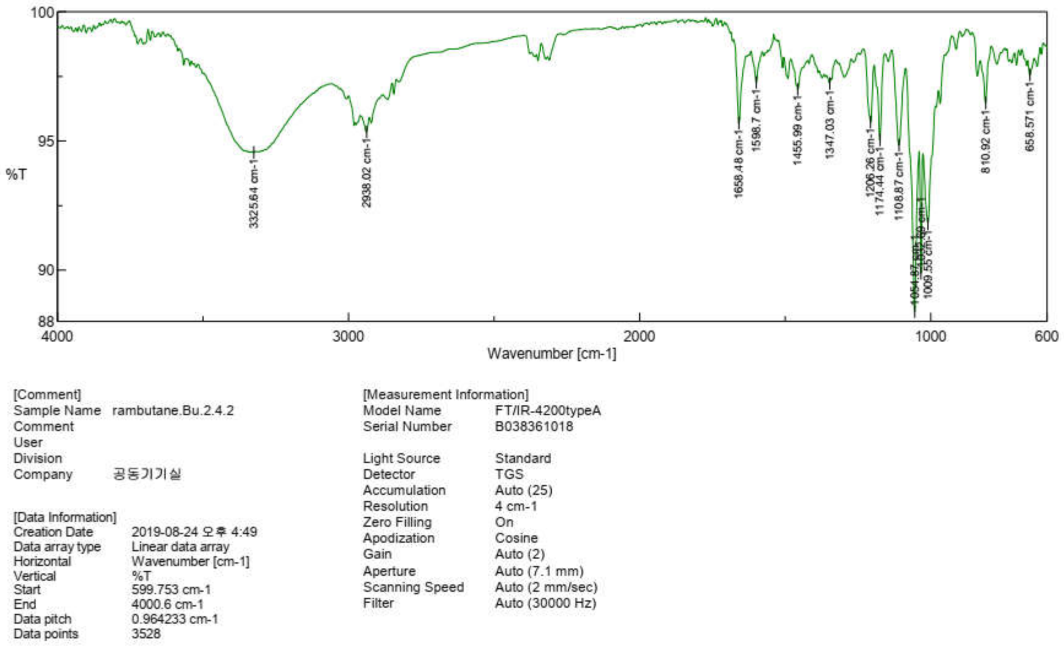

Figure S11: IR spectrum of compound **3.**

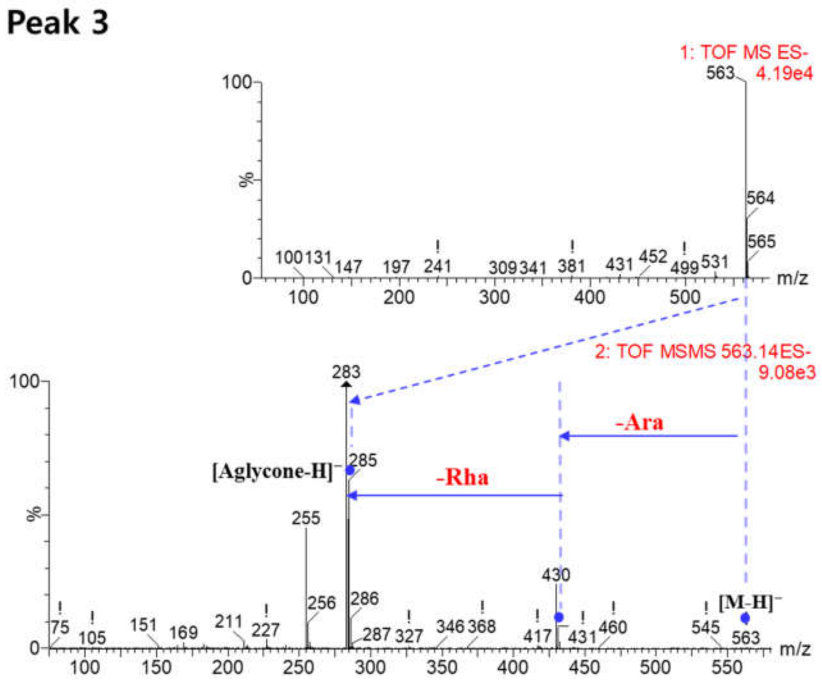

Figure S12: Fragmentation pathway of compound **3.**

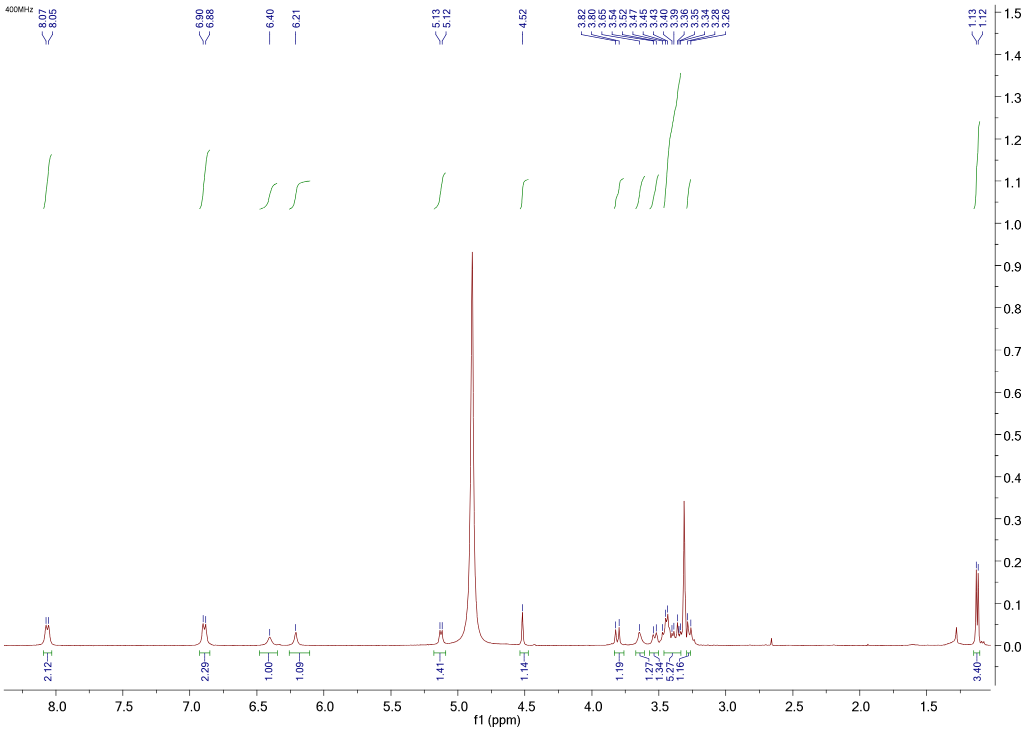

Figure S13: ^{1}H NMR spectrum of compound **4** (400 MHz, Methanol-d_4).

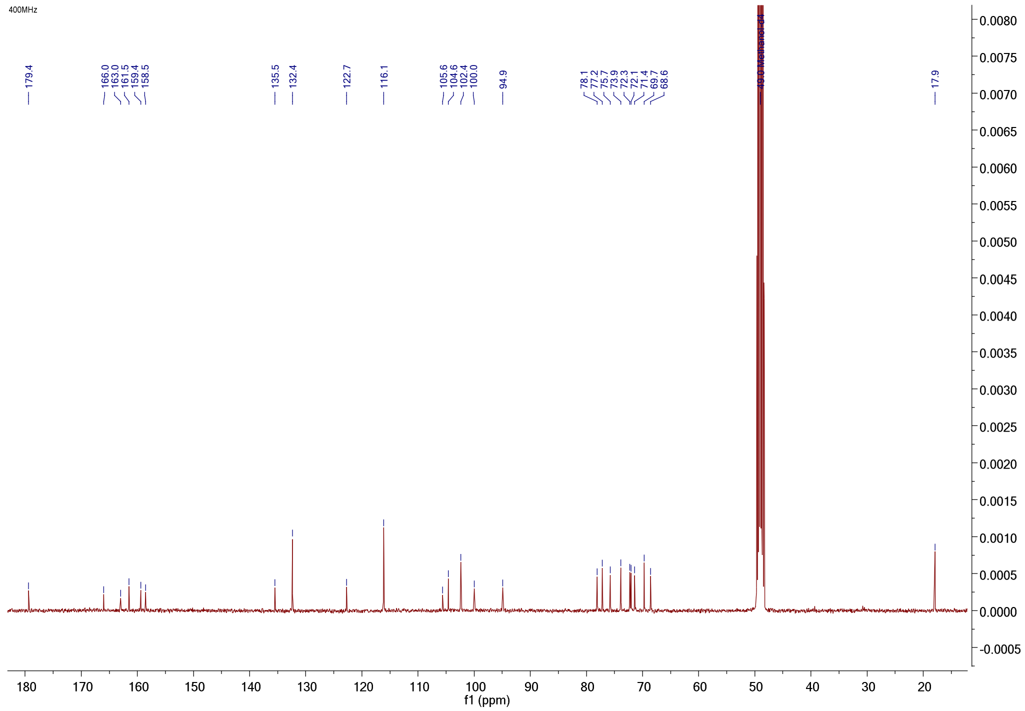

Figure S14: ^{13}C NMR spectrum of compound **4** (100 MHz, Methanol-d_4).

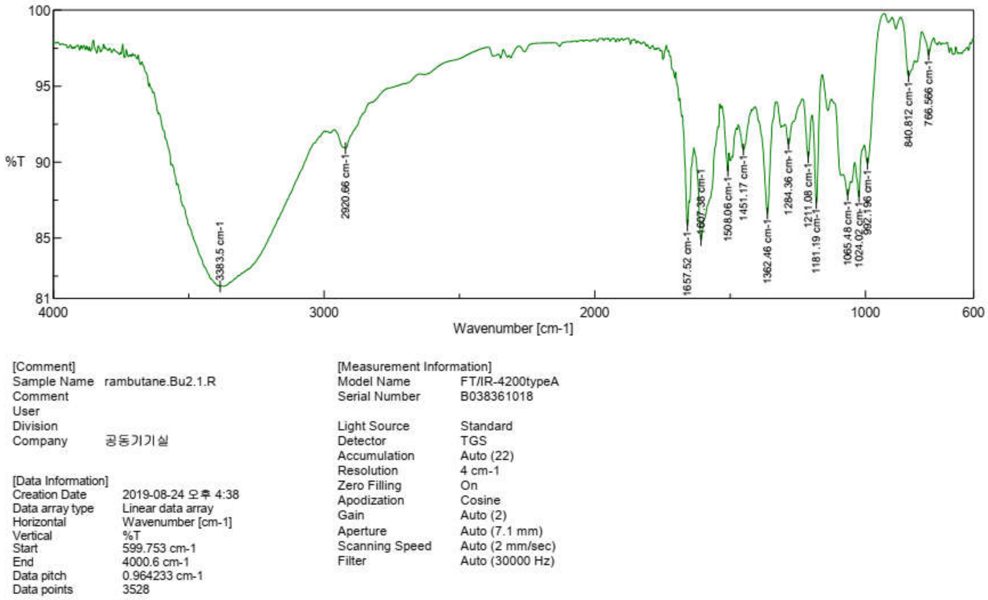

Figure S15: IR spectrum of compound **4.**

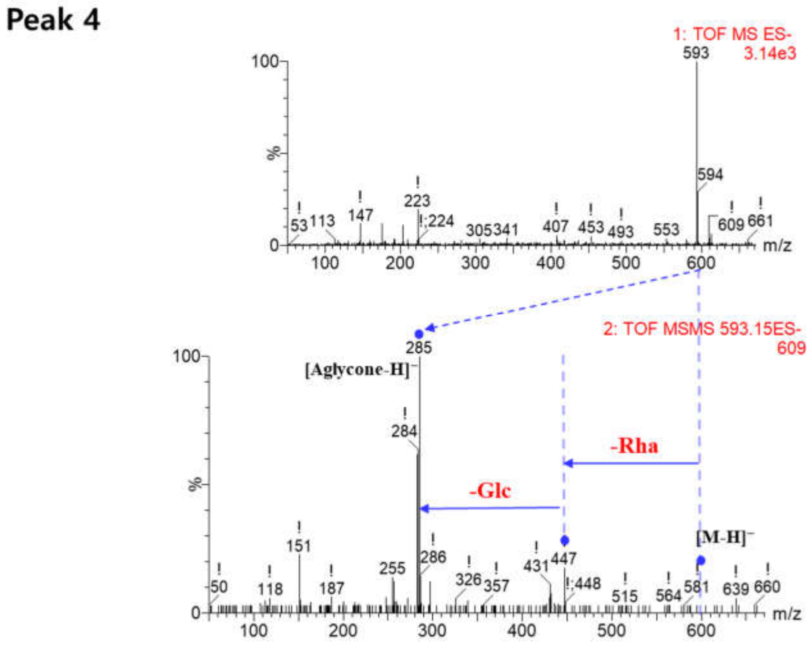

Figure S16: Fragmentation pathway of compound **4.**

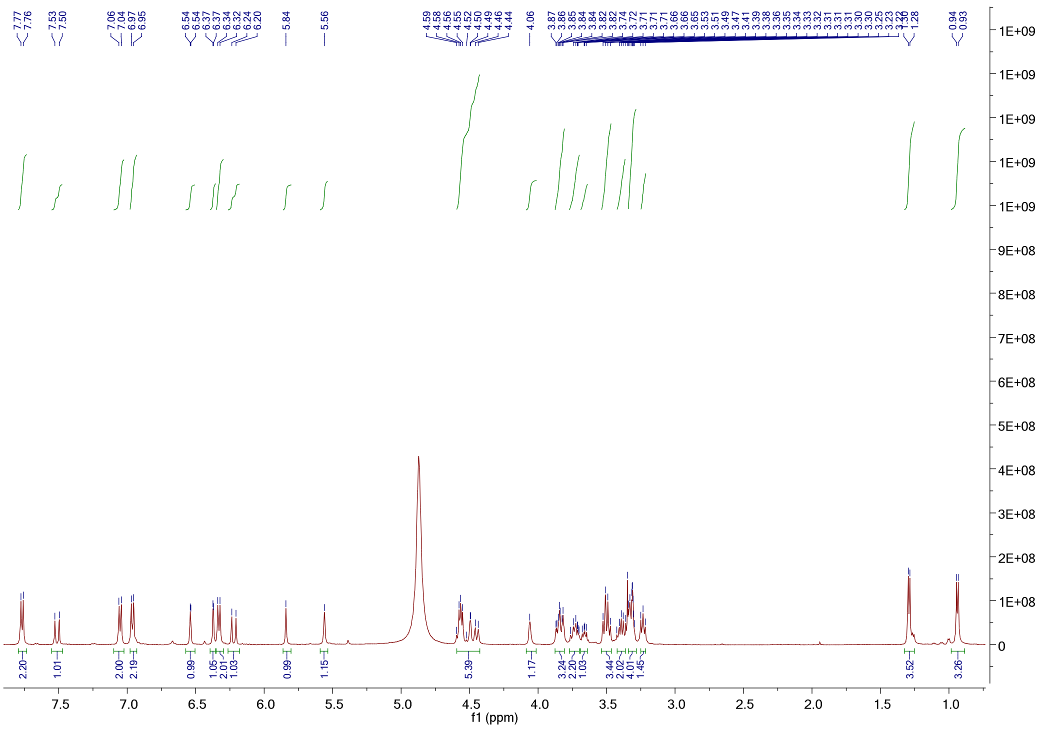

Figure S17:^{1}H NMR spectrum of compound **5** (500 MHz, Methanol-d_4).

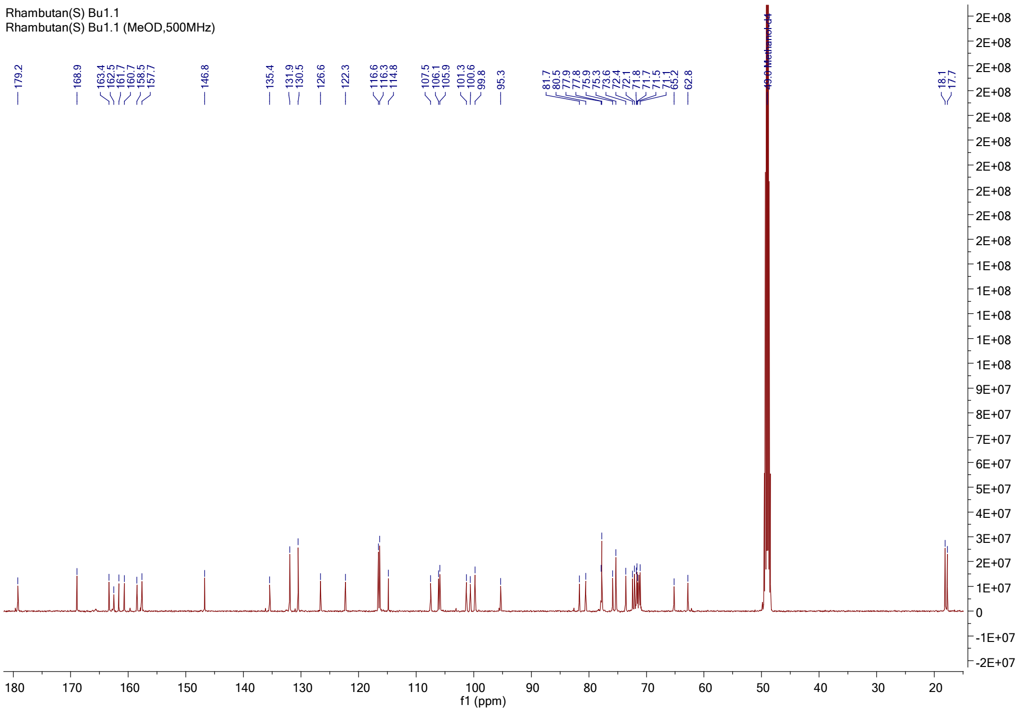

Figure S18:^{13}C NMR spectrum of compound **5** (125 MHz, Methanol-d_4).

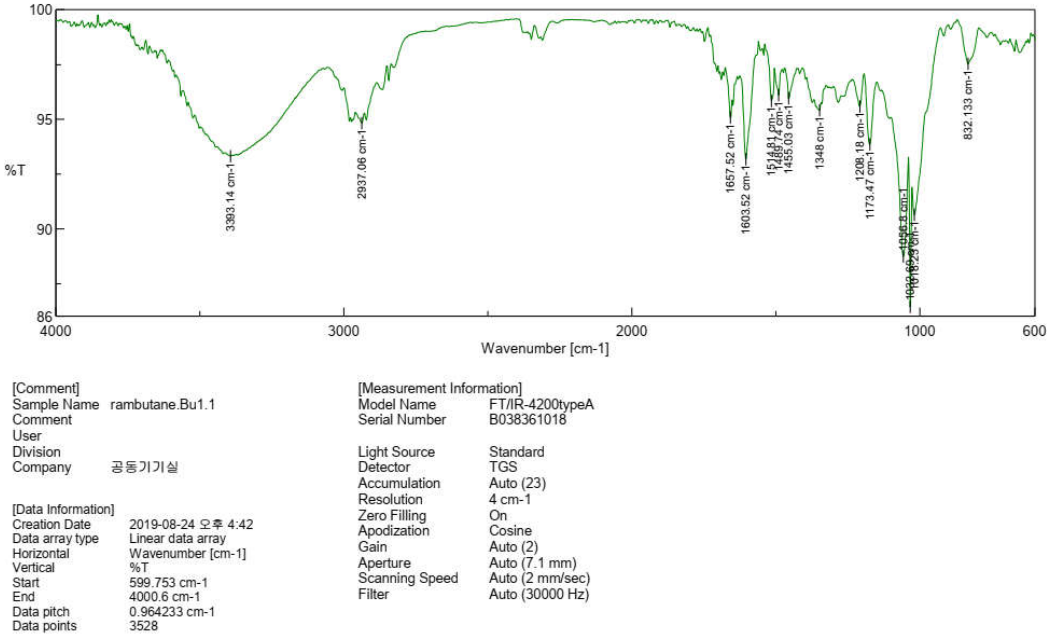

Figure S19: IR spectrum of compound **5.**

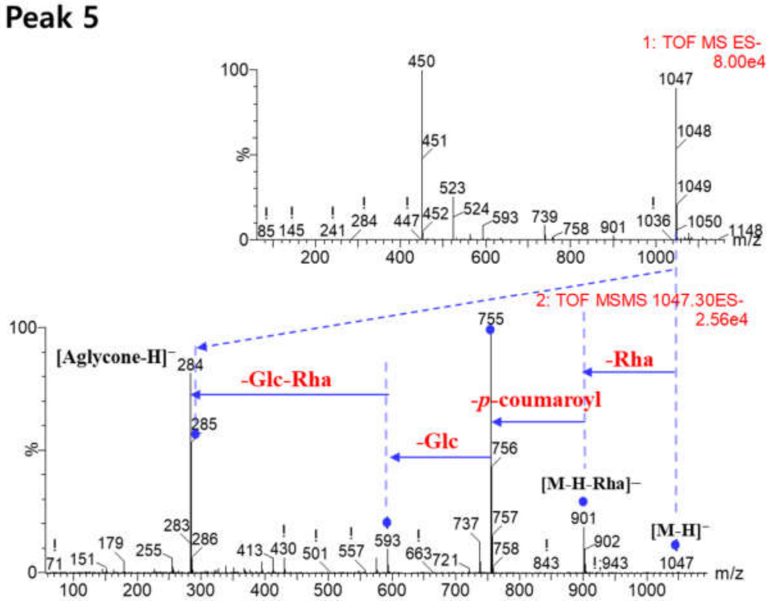

Figure S20: Fragmentation pathway of compound **5.**

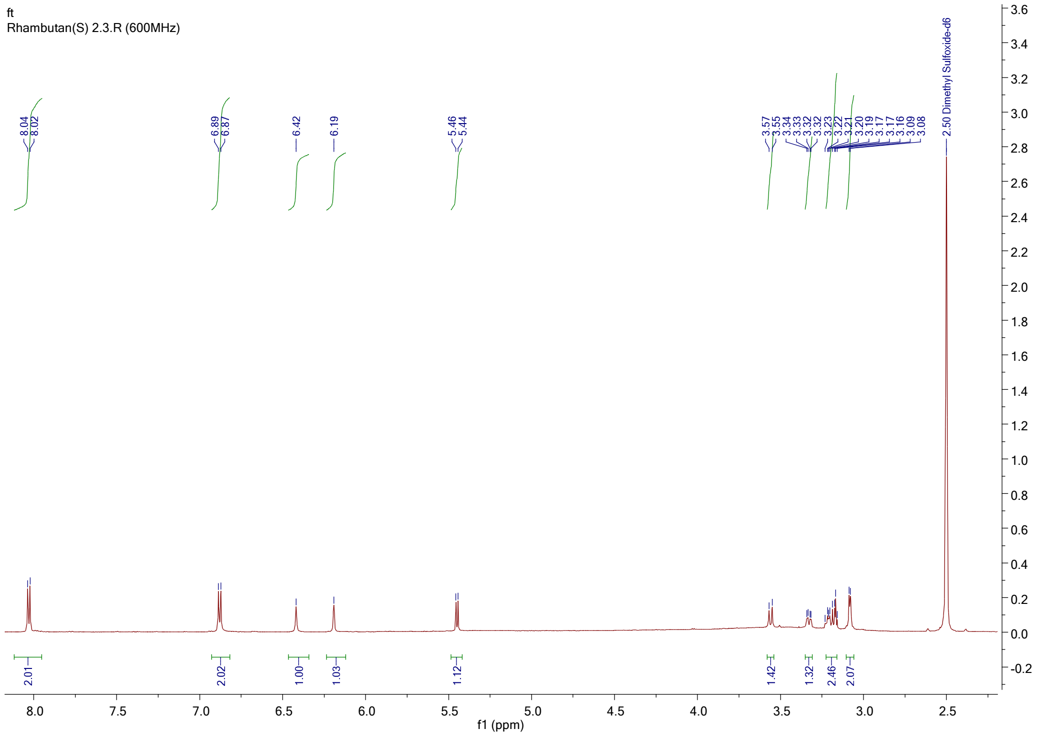

Figure S21:^{1}H NMR spectrum of compound **6** (600 MHz, DMSO-d_6).

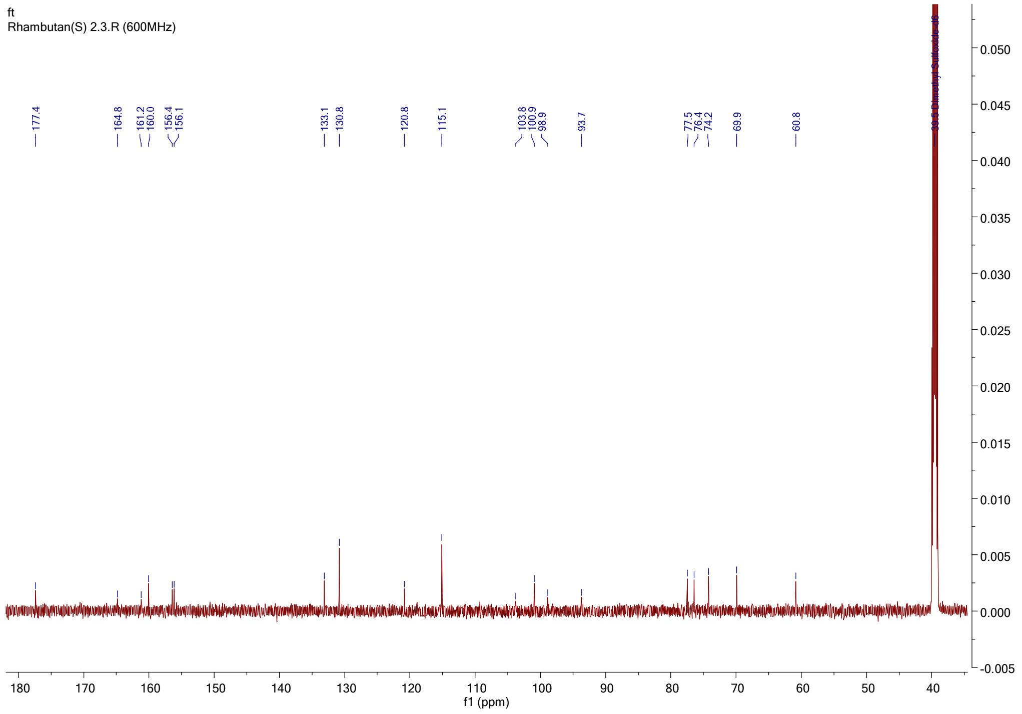

Figure S22:^{13}C NMR spectrum of compound **6** (150 MHz, DMSO-d_6).

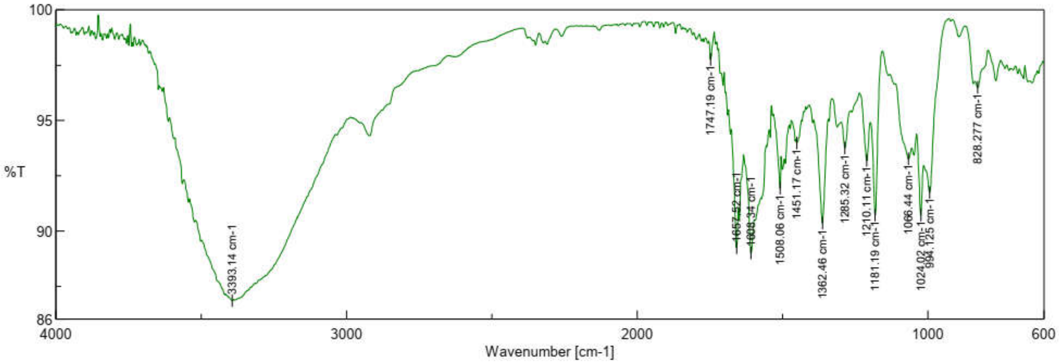

[Comment]
Sample Name rambutane.Bu3.2.R
Comment
User
Division
Company 공동기기실

[Data Information]
Creation Date 2019-08-24 오후 4:34
Data array type Linear data array
Horizontal Wavenumber [cm-1]
Vertical %T
Start 599.753 cm-1
End 4000.6 cm-1
Data pitch 0.964233 cm-1
Data points 3528

[Measurement Information]
Model Name FT/IR-4200typeA
Serial Number B038361018

Light Source Standard
Detector TGS
Accumulation Auto (23)
Resolution 4 cm-1
Zero Filling On
Apodization Cosine
Gain Auto (2)
Aperture Auto (7.1 mm)
Scanning Speed Auto (2 mm/sec)
Filter Auto (30000 Hz)

Figure S23: IR spectrum of compound **6.**

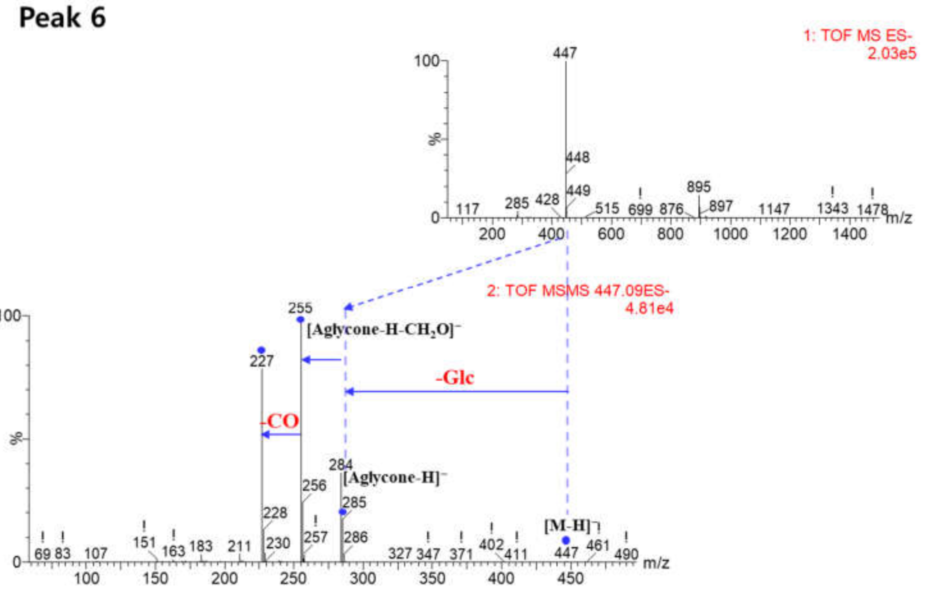

Figure S24: Fragmentation pathway of compound **6.**

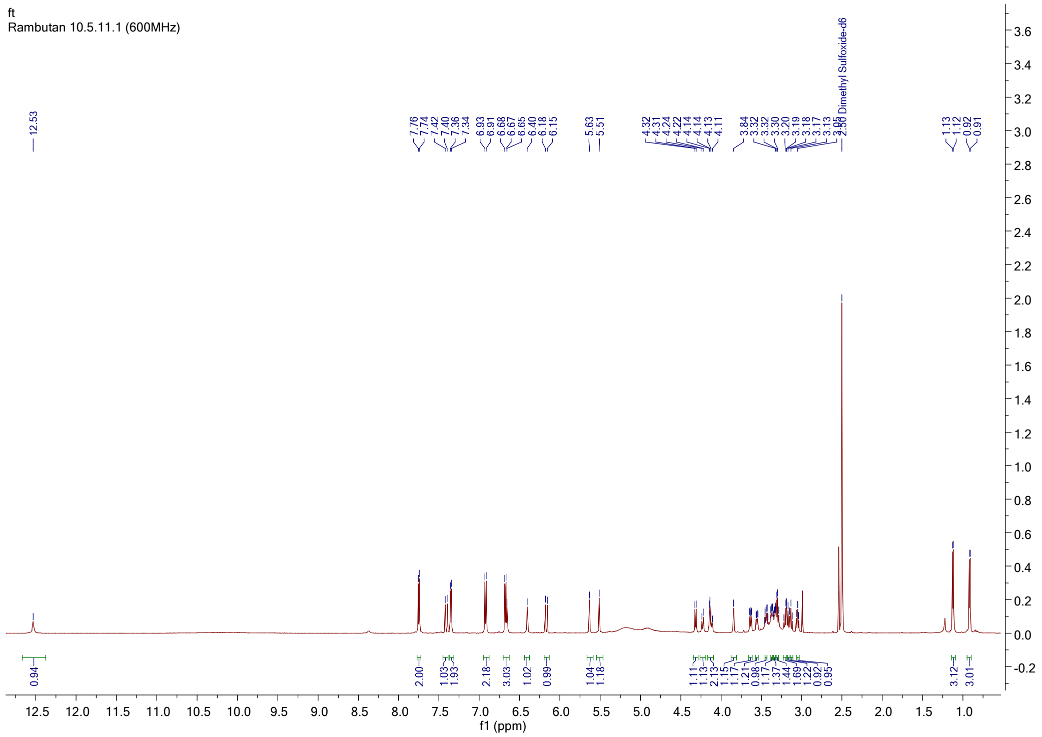

Figure S25:^{1}H NMR spectrum of compound **7** (600 MHz, DMSO-d_6).

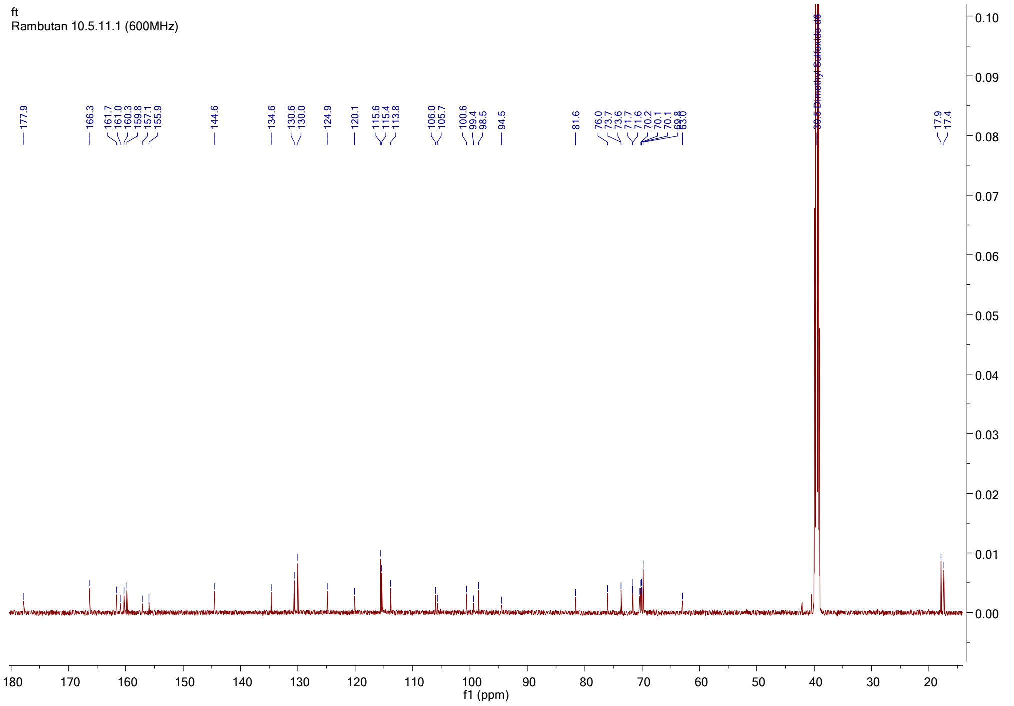

Figure S26:^{13}C NMR spectrum of compound **7** (150 MHz, DMSO-d_6).

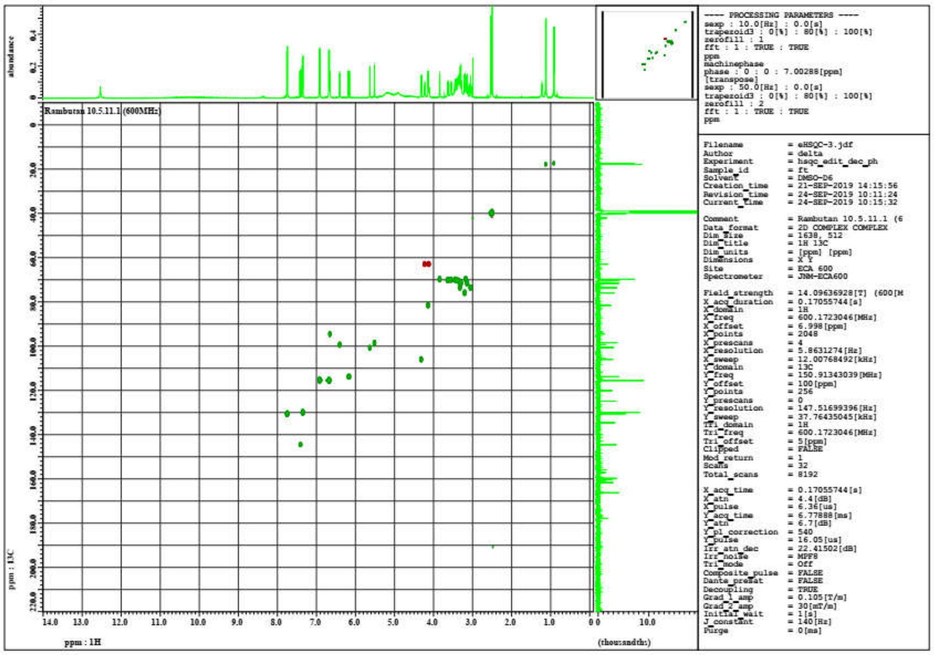

Figure S27: HSQC NMR spectrum of compound **7** (600 MHz, DMSO-d_6)**.**

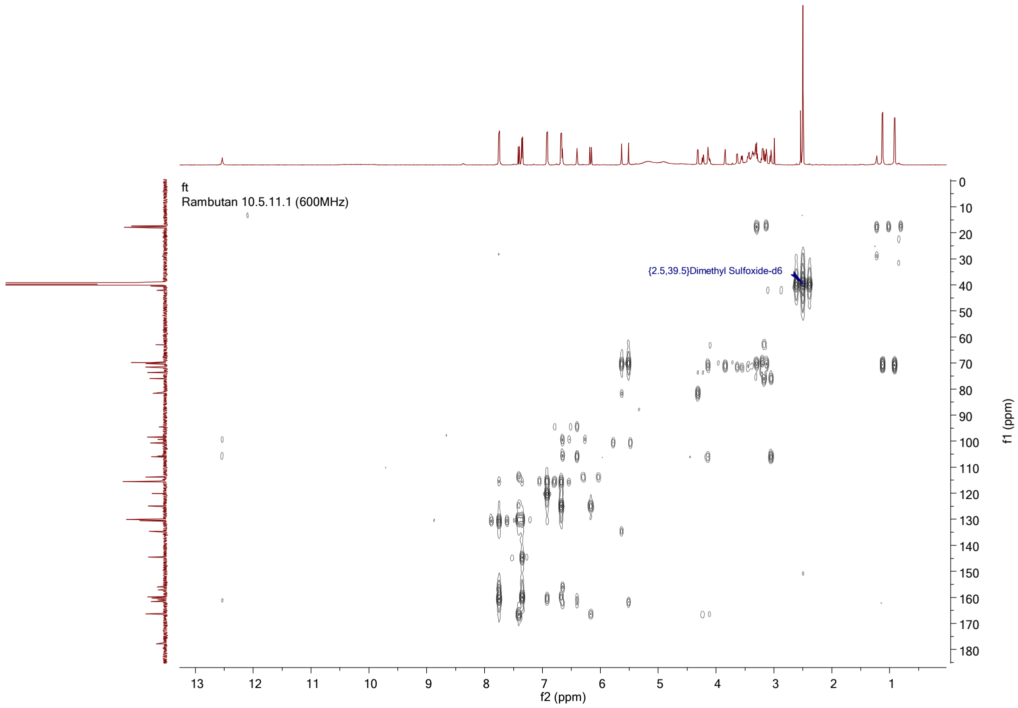

Figure S28: HMBC NMR spectrum of compound **7** (600 MHz, DMSO-d_6).

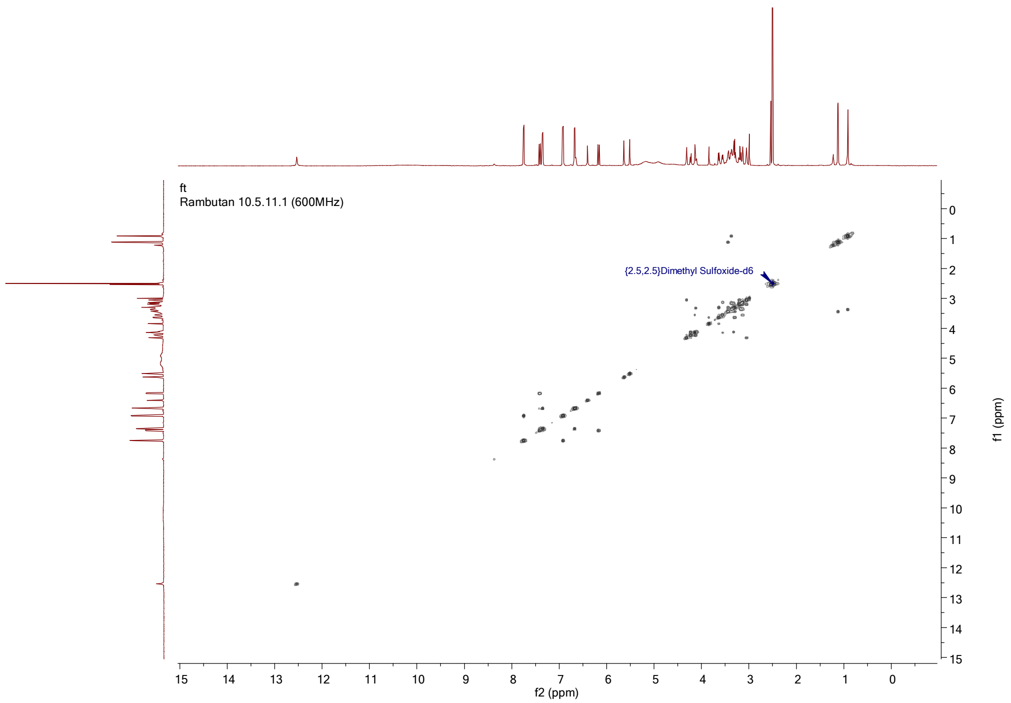

Figure S29: ^{1}H-^{1}H COSY NMR spectrum of compound **7** (600 MHz, DMSO-d_6).

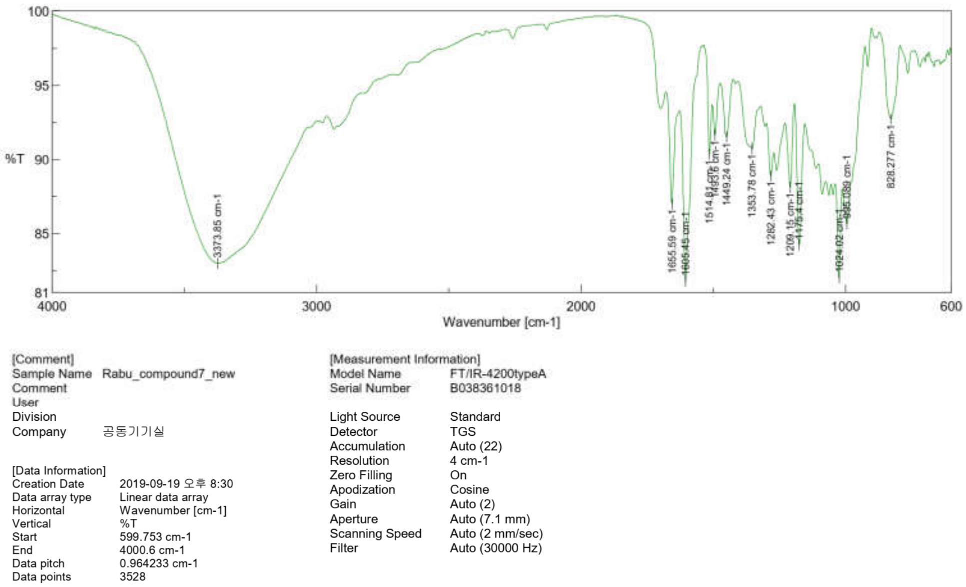

Figure S30: IR spectrum of compound **7.**

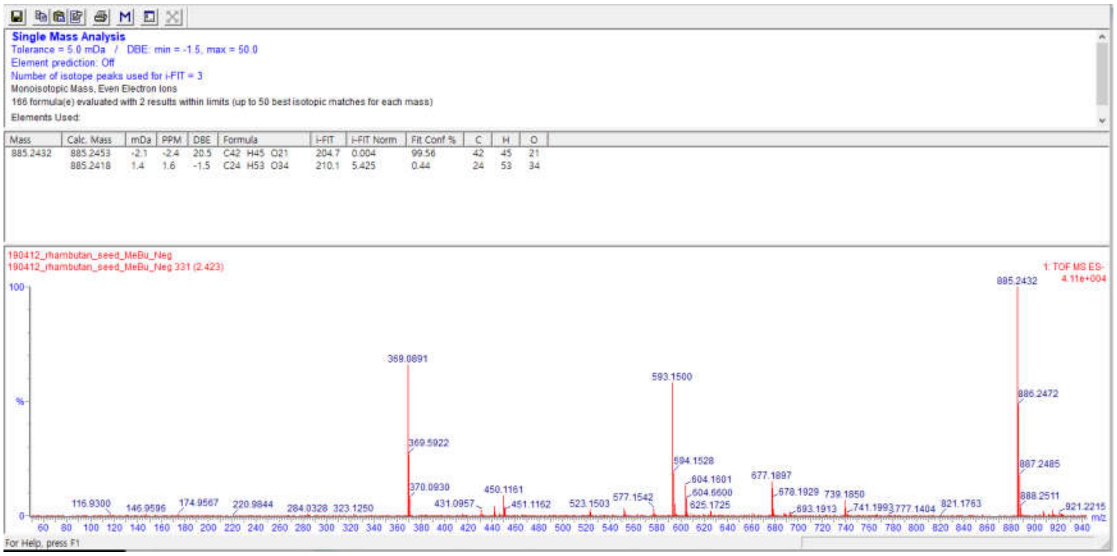

Figure S31: HRESIMS spectrum of compound **7.**

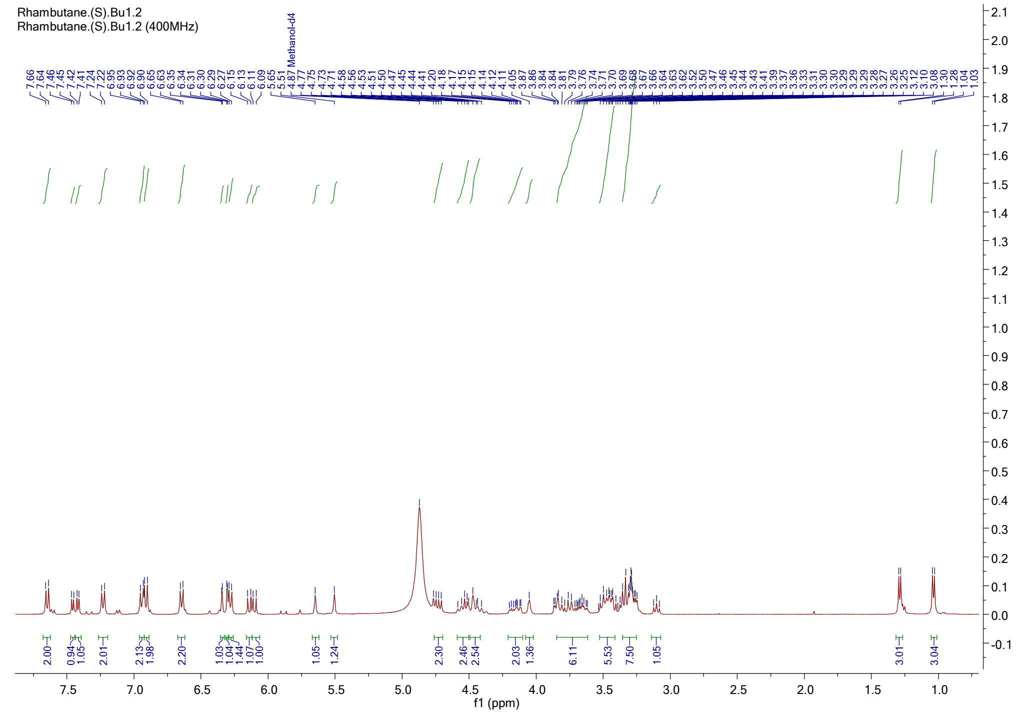

Figure S32:^{1}H NMR spectrum of compound **8** (400 MHz, Methanol-d_4).

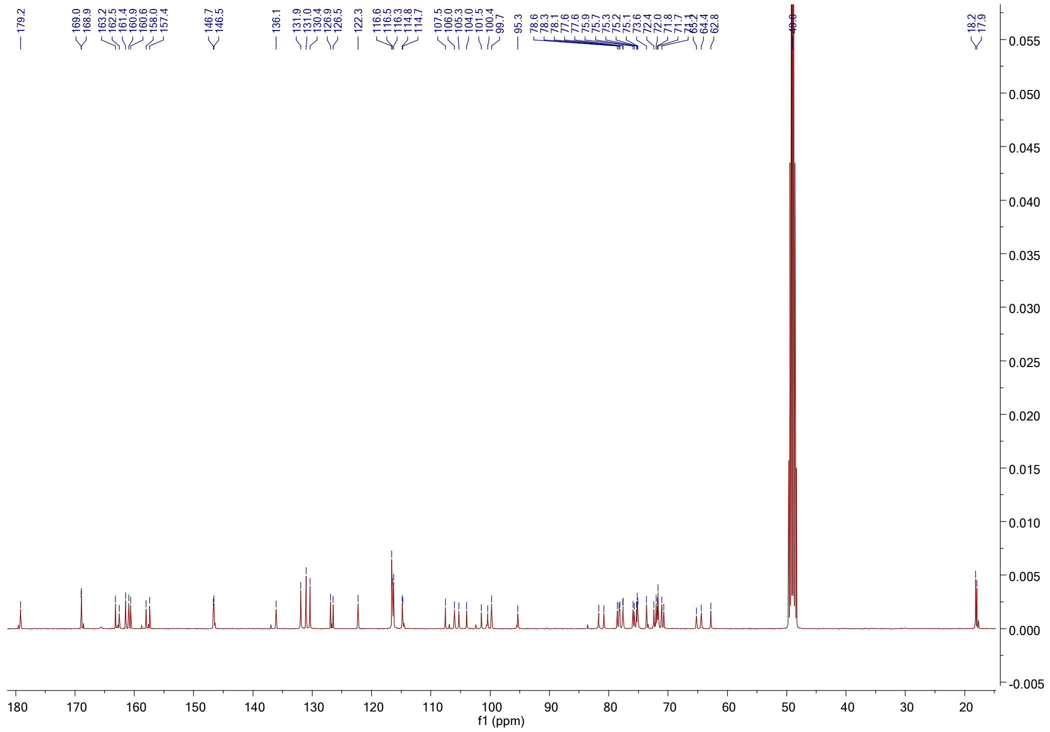

Figure S33:^{13}C NMR spectrum of compound **8** (100 MHz, Methanol-d_4).

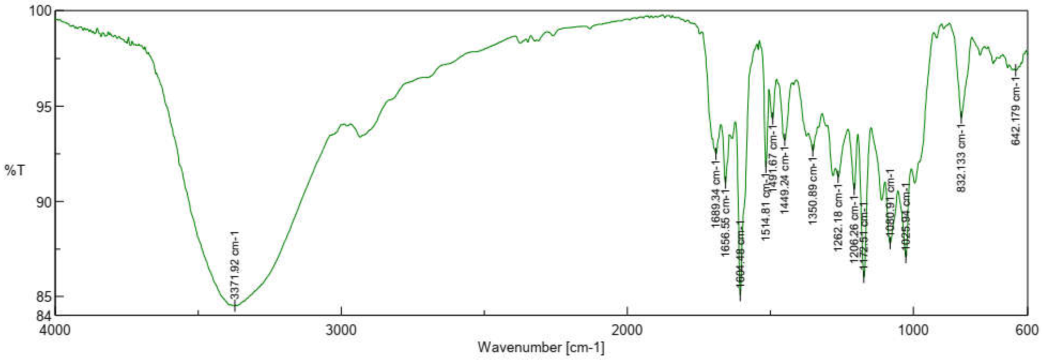

[Comment]
Sample Name rambutane.Bu1.2
Comment
User
Division
Company 공동기기실

[Data Information]
Creation Date 2019-08-24 오후 4:23
Data array type Linear data array
Horizontal Wavenumber [cm-1]
Vertical %T
Start 599.753 cm-1
End 4000.6 cm-1
Data pitch 0.964233 cm-1
Data points 3528

[Measurement Information]
Model Name FT/IR-4200typeA
Serial Number B038361018

Light Source Standard
Detector TGS
Accumulation Auto (23)
Resolution 4 cm-1
Zero Filling On
Apodization Cosine
Gain Auto (2)
Aperture Auto (7.1 mm)
Scanning Speed Auto (2 mm/sec)
Filter Auto (30000 Hz)

Figure S34: IR spectrum of compound **8.**

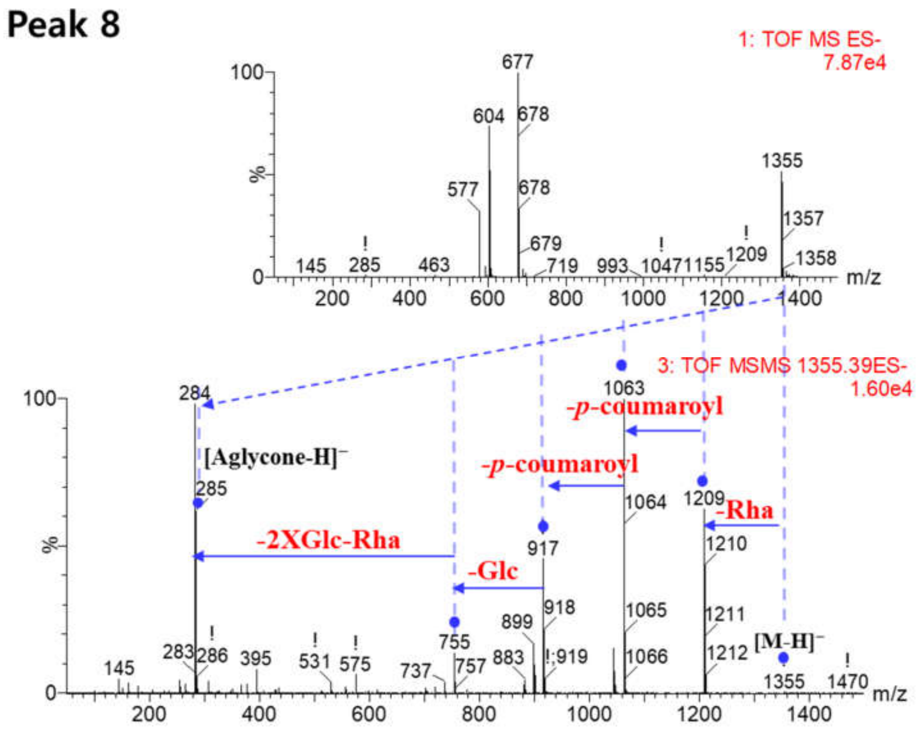

Figure S35: Fragmentation pathway of compound **8.**

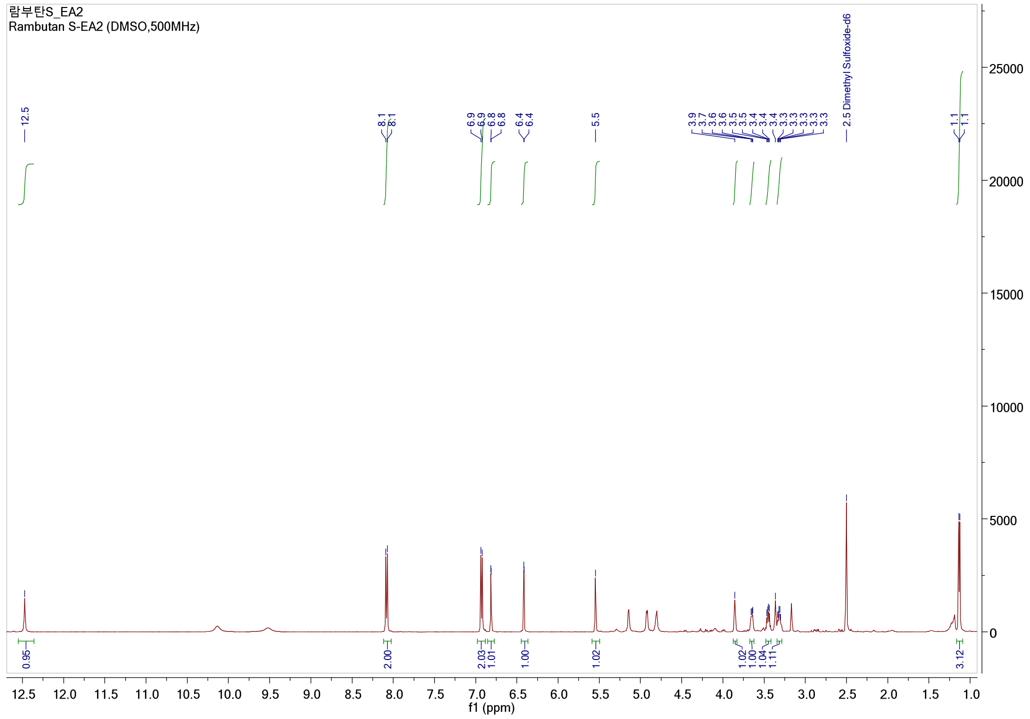

Figure S36:^{1}H NMR spectrum of compound **9** (500 MHz, DMSO-d_6).

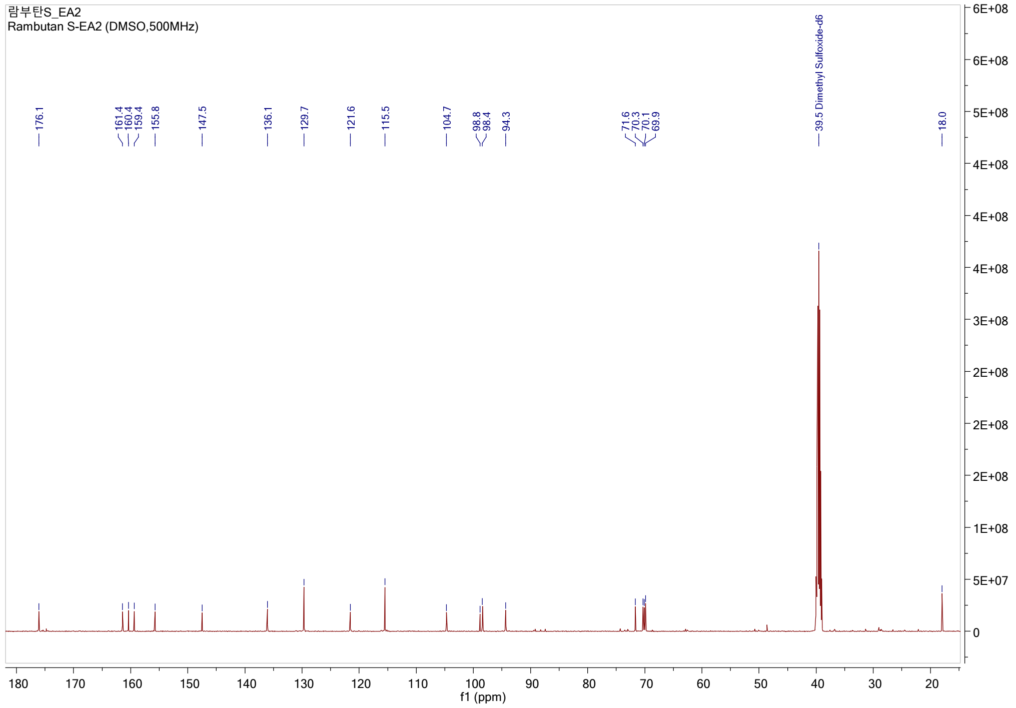

Figure S37:^{13}C NMR spectrum of compound **9** (125 MHz, DMSO-d_6).

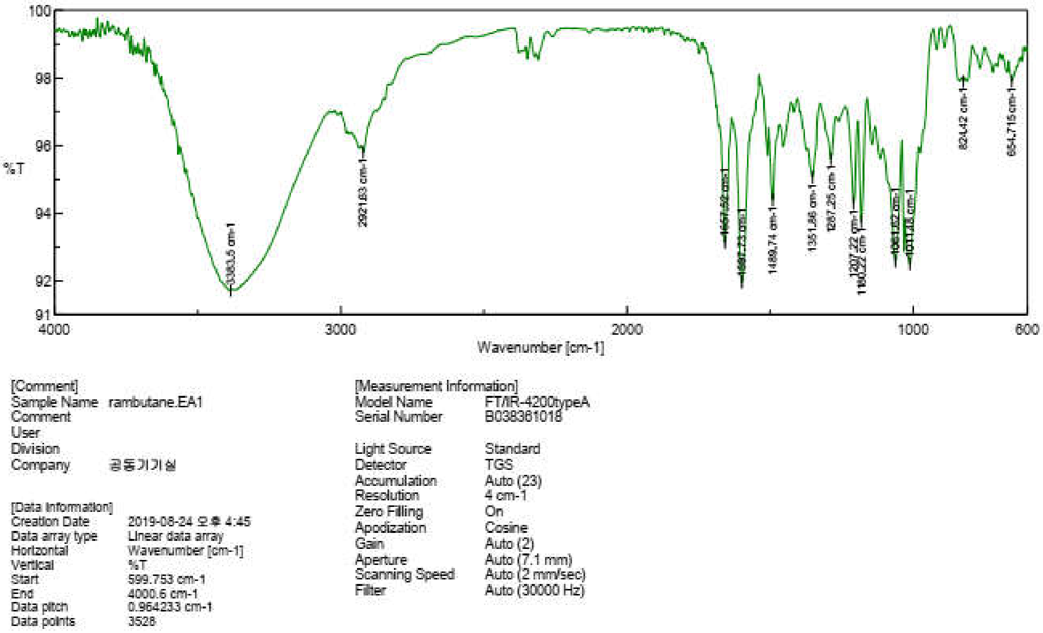

Figure S38: IR spectrum of compound **9.**

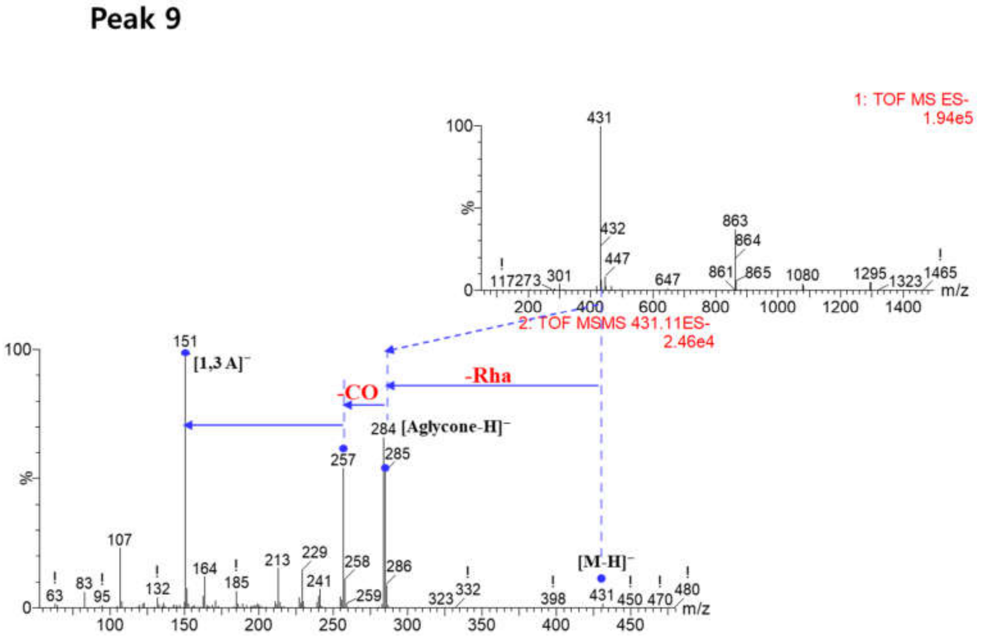

Figure S39: Fragmentation pathway of compound **9.**

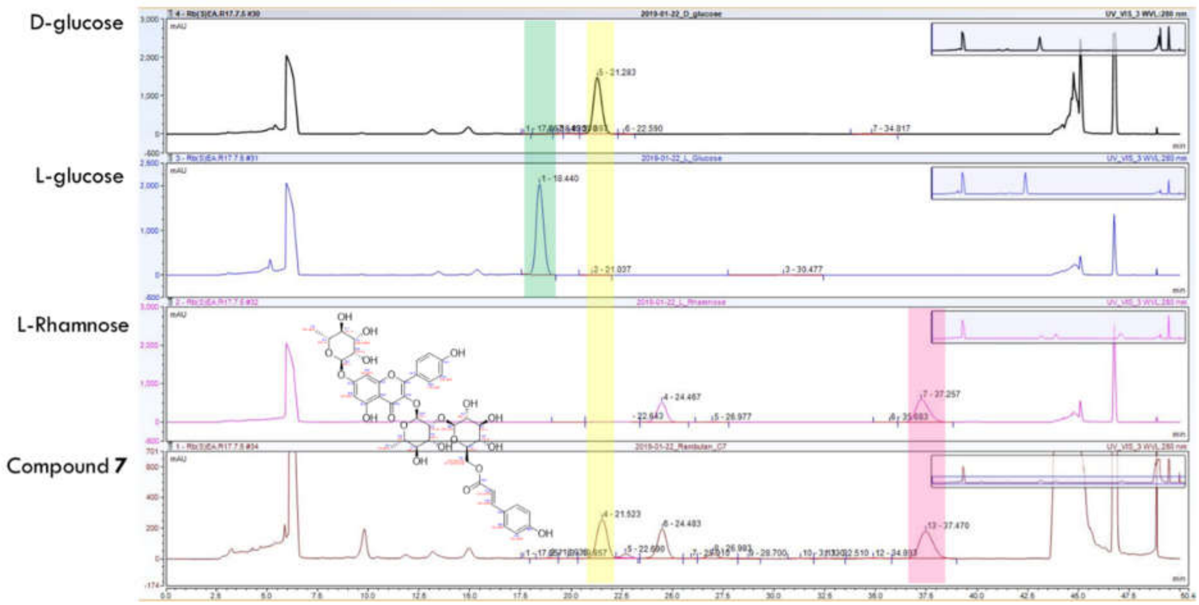

Figure S40: Sugar Analysis. Compound **7** (2.5 mg) was heated in 0.1 mL at 110°C for 1.5 h of 2 M HCl. After acid hydrolysed sample was neutralized with Na_2CO_3 and dried. The residue was derivatized with L-cysteine methyl ester hydrochloride in anhydrous pyridine (200 μL, 60 °C, 1 h) and subsequently added phenylisothiocyanate (1 μL, 60 °C, 1 h). The sugar derivatives from compound **7** were compared with standard sugar derivatives by the HPLC analysis and were confirm the absolute configuration of these sugars to be D-glucose, and L-rhamnose.

Figure S41: Molecular Networking

HRESIMS / MS data of the bioactive fractions of N. *lappaceum* seed and extracts of each part of *N. lappaceum* (pulp, peel, seed) were performed by Xevo G2 QTOF mass spectrometer with a Waters Acquity UHPLC® BEH C18 (100 mm × 2.1 mm, 1.7 μm) column. Those data generated molecular networking job with HRESIMS/MS spectra and analyzed in comparison with several reference papers and advanced library searches of GNPS. Before creating molecular networking, MS / MS data were preprocessed using the MZmine 2 program to increase reliability and then uploaded on the GNPS platform (https://gnps.ucsd.edu) to create molecular networking. The parameters for the generation of the molecular network were set as follows: the precursor ion mass tolerance of 0.02 Da, Fragment ion mass tolerance of 0.02 Da, a cosine score above 0.65, minimum matched fragment ions below 4 counts were removed from the MS/MS spectra. The Matching with the publicly accessible GNPS spectral library was performed with a score threshold of 0.65 and matched fragment ion number above 4. After that, the GNPS data were visualized using Cytoscape 3.7.1. The completed molecular network of *N. lappaceum* active fractions is explored and downloaded from the GNPS website via the following link.: https://gnps.ucsd.edu/ProteoSAFe/status.jsp?task=301c3a87d4594d648031a1399c2c3ff7. The completed molecular network of *N. lappaceum* pulp, peel, seed extract is explored and downloaded from the GNPS website via the following link: https://gnps.ucsd.edu/ProteoSAFe/status.jsp?task=9b427c2e16304de89f32167f679612f1.

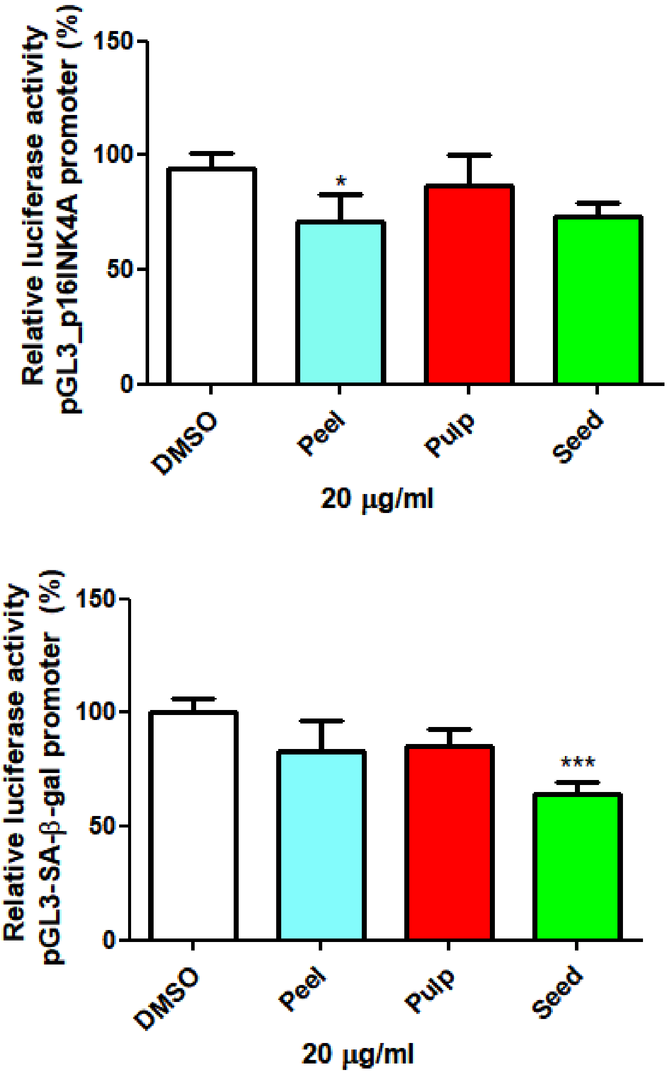

Figure S42: Effect of *N. lappaceum* peel, pulp and seed on p16Ink4A and SA-β-gal promoter activity in human dermal fibroblasts.

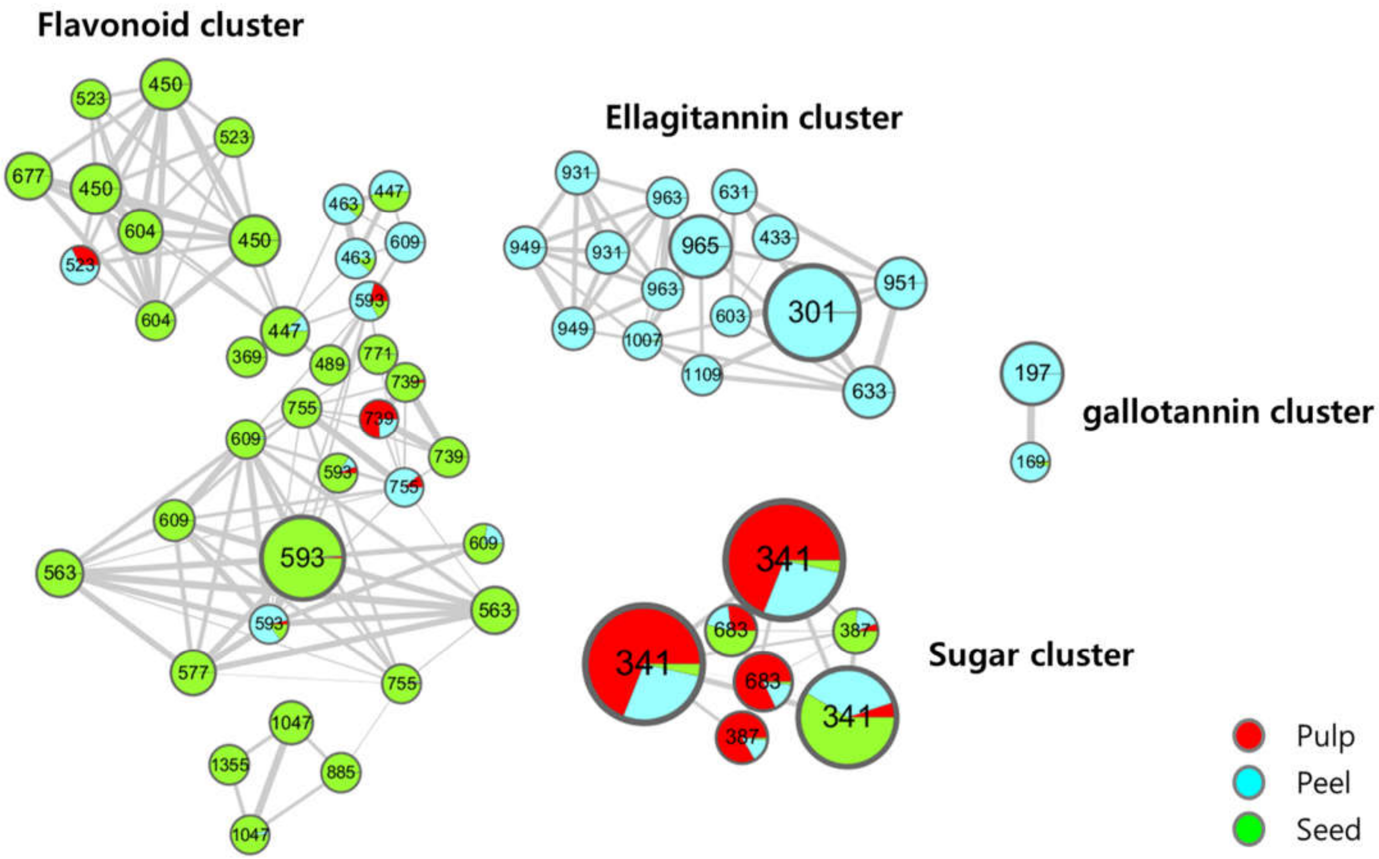

Figure S43: Annotation of the molecular networking of the crude extract from *N. lappaceum* pulp (red nodes), peel (Aquamarine nodes), seed (green nodes) which shows flavonoid, ellagitannin, gallotannin and sugar.

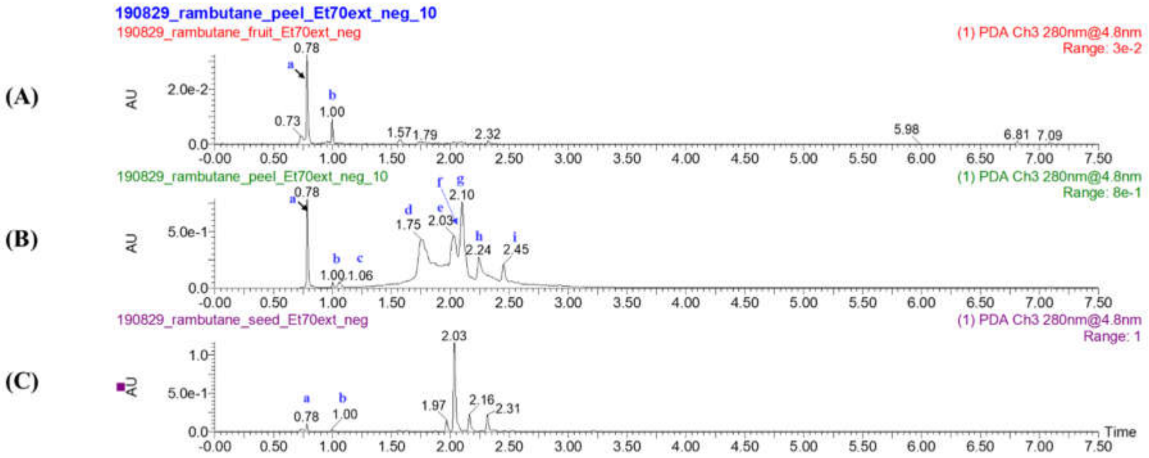

(D)

Peak	t_R(mins)	Compounds	Formula [M−H]$^-$	[M−H]$^-$ Observed	[M−H]$^-$ Calculated	MS/MS fragments	Error (ppm)	Group	References
a	0.79	Disaccharides	$C_{12}H_{21}O_{11}$	341.1092	341.1084	179,161	2.3	Sugar	(Verardo et. al., 2009) (Gabbanini et. al., 2010)
b	0.99	Disaccharides	$C_{12}H_{21}O_{11}$	341.1087	341.1084	179,161	0.9	Sugar	(Verardo et. al., 2009) (Gabbanini et. al., 2010)
c	1.06	Gallic acid	$C_7H_5O_5$	169.0143	169.0137	125	3.5	Gallotannins	(Yisimayili et. al., 2019) (Sun et. al., 2014) (Mena et. al., 2012)
d	1.75	Corilagin	$C_{27}H_{21}O_{18}$	633.0724	633.0728	463, 301, 279, 169	-0.6	Ellagitannin	(Yisimayili et. al., 2019) (Hernández et. al., 2017) (Thitilertdecha et. al., 2010)
e	2.03	Geraniin	$C_{41}H_{27}O_{27}$	951.0709	951.0740	933, 301, 169	-3.3	Ellagitannin	(Hernández et. al., 2017) (Thitilertdecha et. al., 2010) (Thitilertdecha et. al., 2010)
f	2.05	Ellagic acid pentoside	$C_{19}H_{13}O_{12}$	433.0406	433.0407	301, 169	-0.2	Ellagitannin	(Hernández et. al., 2017)
g	2.10	Rhoipteleanina H	$C_{42}H_{29}O_{27}$	965.0887	965.0896	301, 247, 169	-0.9	Ellagitannin	(Jiang et. al., 1999) (Quintela et. al., 2017)
h	2.24	Ellagic acid	$C_{14}H_5O_8$	300.9998	300.9984	257, 229, 185	4.7	Ellagitannin	(Yisimayili et. al., 2019) (Mena et. al., 2012) (Hernández et. al., 2017) (Thitilertdecha et. al., 2010)
i	2.45	ethyl gallate	$C_9H_9O_5$	197.0445	197.0450	169, 124	-2.5	gallotannins	(Zhang et. al., 2017) (Mahmood et. al., 2018)

Figure S44: DAD chromatograms of crude extract of *N. lappaceum* pulp (A), peel (B), and seed (C) at 280 nm. (D) major compounds of pulp, peel and seed extracts of *N. lappaceum* identified in corresponding MS / MS fragmentation profiles.

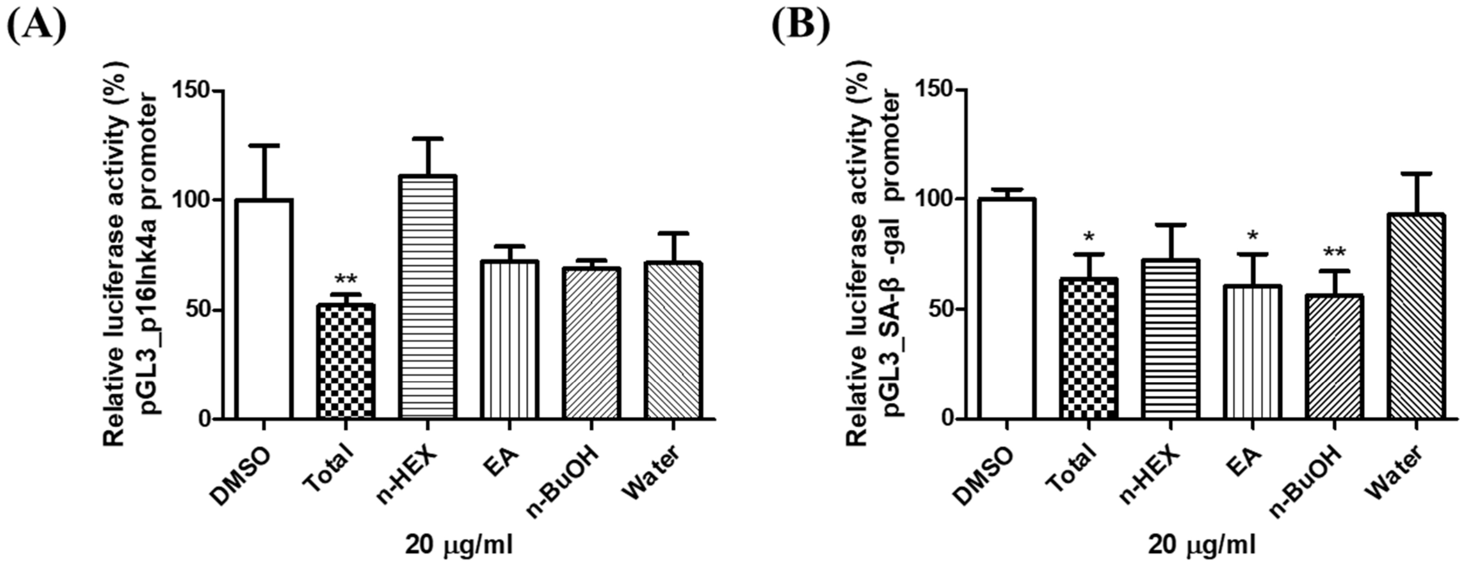

Figure S45. Effect of crude extract and four fractions from *N. lappaceum* seeds on p16INK4A and SA-β-gal transcription in human dermal fibroblasts. Human dermal fibroblasts were transiently co-transfected with pGL3-p16ink4a (**A**) or pGL3-glb1 (**B**) promoter with β-galactosidase as a transfection control.

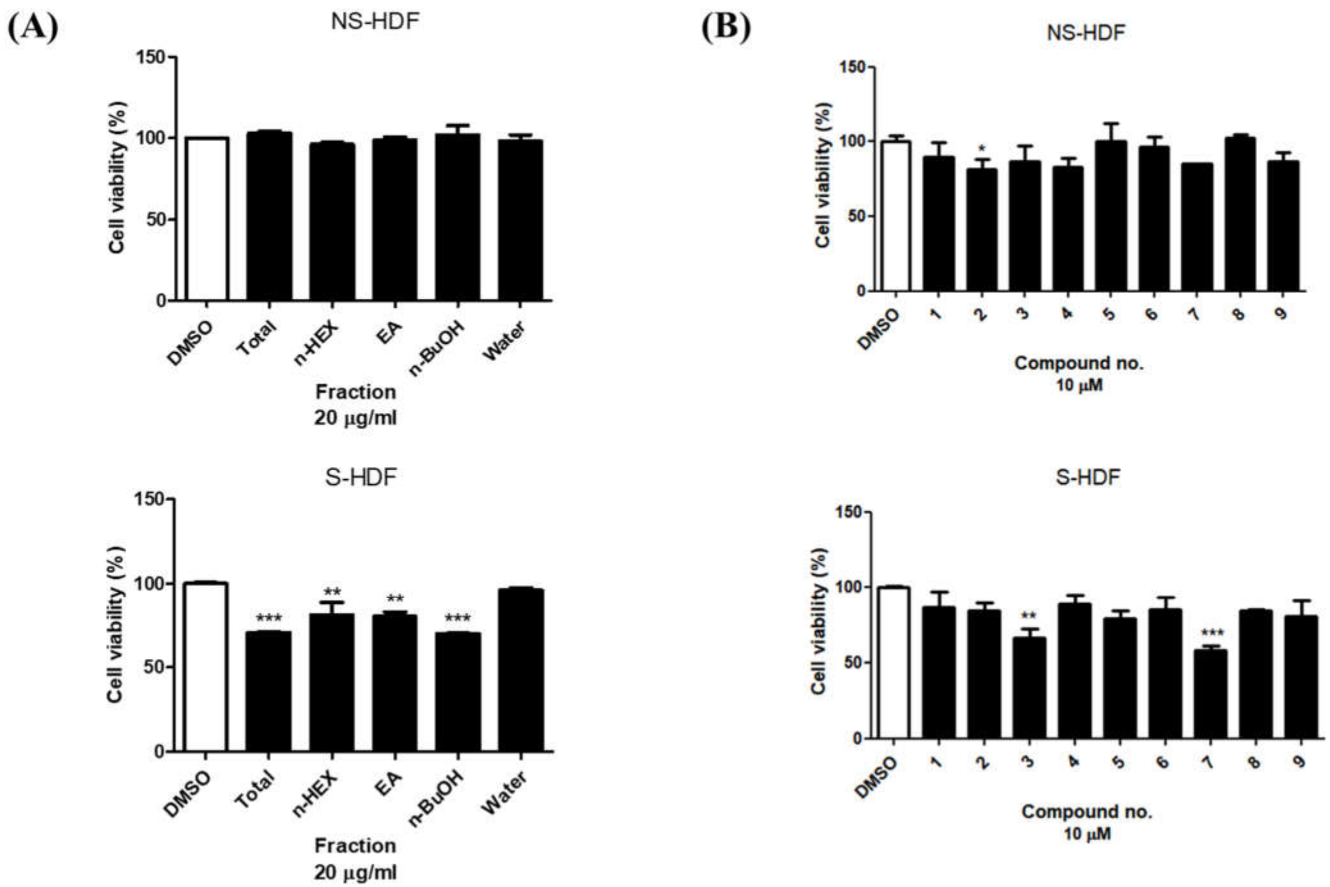

Figure S46: The cytotoxicity effect of total extract and four fractions 20 μg/mL (A) and 10 μM compounds **1-9** obtained from *N. lappaceum* seeds (B) both in young and senescent HDFs. After 72 h of incubation with tested fractions and compounds, the MTT assay was carried out as described in the experimental section.

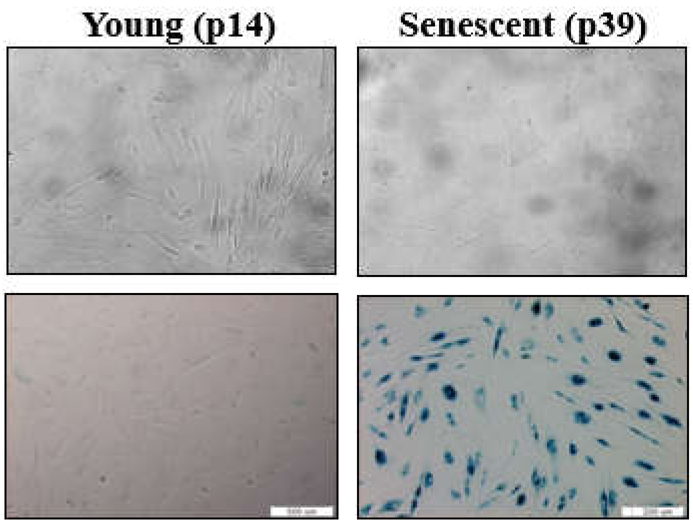

Figure S47: Senescence-associated β-galactosidase staining of HDFs. Young and aged HDFs stained for 24h for SA-β galactosidase. Images of young HDFs (passage 14) had little staining, while aged HDFs (passage 39) stained blue.

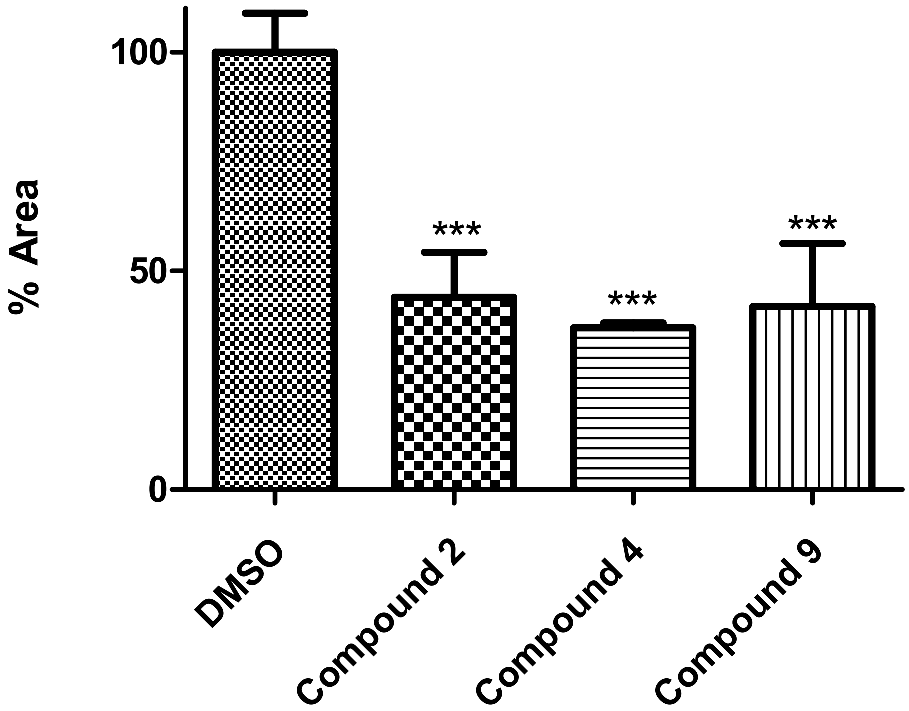

Figure S48: Quantification of senescence-associated β-galactosidase staining. Aged HDF cells were treated three times over 6 days with vehicle or 10 μM compounds **2**, **4**, and **9** and quantified through images obtained from a fluorescence microscope. Data were calculated by the mean ±SD of three independent experiments. Significant differences between the groups were determined using one-way analysis of variance (ANOVA) with the Tukey method. Statistical significance was accepted at * $p < 0.05$, ** $p < 0.01$, and *** $p < 0.001$.

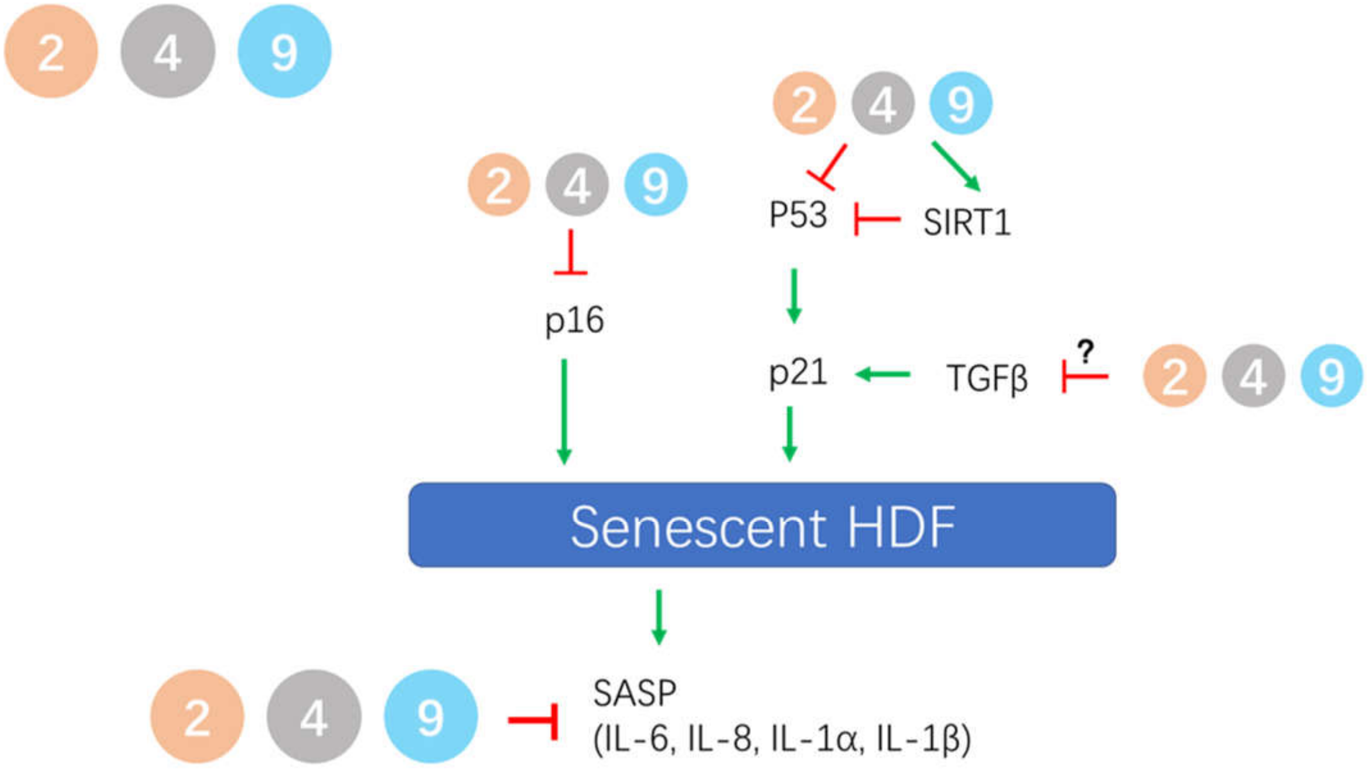

Figure S49: Mechanism of action of compounds **2**, **4**, and **9** isolated from Rambutan.

Table S1. ^{1}H NMR data for compounds **1**–**6, 8 and 9**.

position	**1**[a]	**2**[b]	**3**[a]	**4**[c]	**5**[d]	**6**[a]	**8**[c]	**9**[b]
	δ_H, mult (*J* in Hz)	δ_H, mult (*J* in Hz)	δ_H, mult (*J* in Hz)	δ_H, mult (*J* in Hz)	δ_H, mult (*J* in Hz)	δ_H, mult (*J* in Hz)	δ_H, mult (*J* in Hz)	δ_H, mult (*J* in Hz)
**6**	6.44, s	6.44, d (1.7)	6.45, s	6.21, s	6.37, d (1.8)	6.19, br s	6.31, d (2.0)	6.82, d (2.0)
**8**	6.82, s	6.83, d, (1.7)	6.83, s	6.40, s	6.54, d (1.8)	6.42, br s	6.35, d (2.0)	6.42, d (2.0)
**2′**	8.11, d (8.8)	8.07, d (8.8)	8.12, d (8.8)	8.05, d (7.7)	7.77, br d (8.7)	8.03, d (8.7)	7.64, d (8.7)	8.09, d (8.9)
**3′**	6.87, d (8.8)	6.88, d (8.8)	6.89, d (8.8)	6.89, d (7.7)	6.95, br d (8.7)	6.88, d (8.7)	6.90, d (8.7)	6.94, d 8.9)
**5′**	6.87, d (8.8)	6.88, d (8.8)	6.89, d (8.8)	6.89, d (7.7)	6.95, br d (8.7)	6.88, d (8.7)	6.90, d (8.7)	6.94, d 8.9)
**6′**	8.11, d (8.8)	8.07, d (8.8)	8.12, d (8.8)	8.05, d (7.7)	7.77, br d (8.7)	8.03, d (8.7)	7.64, d (8.7)	8.09, d (8.9)
	3-Galactosyl	***3-Glucosyl***	***3-arabinosyl***	***3-Glucosyl***	***3-Rhamnosyl***	***3-Glucosyl***	***3-Rhamnosyl***	
**1″**	5.44, d (7.6)	5.47, d (7.3)	5.36, d (5.1)	5.13, d (6.6)	5.84, s	5.46, d (7.5)	5.65, brs	
**2″**	3.17-3.85, m[e]	3.08-4.29, m[e]	3.75, t (5.5)	3.3-3.6[e]	4.49[e]	3.19[e]	4.47, m[e]	
**3″**			3.53, dd (6.8, 3.0)		3.84[e]	3.21[e]	4.11, dd, (2.7,10.0)	
**4″**			3.65, m[e]		3.49[e]	3.09[e]	3.75, m[e]	
**5″**			3.58, dd, (11.5, 5.5)		3.23[e]	3.09[e]	3.49-3.36, m[e]	
			3.21, dd (11.4, 2.5)					
**6″**				3.80, br d (10.1)	0.94, d (6.1)	3.57, d (11.5)	1.03 (d, J = 6.1) [e]	
				3.3-3.6[e]		3.33, d (11.4, 4.2)		
	7-Rhamnosyl	***7-Rhamnosyl***	***7-Rhamnosyl***	***6''-Rhamnosyl***	***7-Rhamnosyl***		***7-Rhamnosyl***	***7-Rhamnosyl***
**1‴**	5.55, s	5.55, s	5.55, s	4.5, d (1.5)	5.56, s		5.51, br s	5.55, s
**2‴**	3.17-3.85, m	3.08-4.29, m[e]	3.84, br s	3.65, br s	4.06, br s		4.05, br s	3.85, br s
**3‴**			3.64, dd (9.3, 3.2)	3.51, d (9.0)	3.86[e]		3.83, m[e]	3.66, m
**4‴**			3.30[e]	3.3-3.6[e]	3.49[e]		3.48, m[e]	3.31, m

**5′′′**			3.43, dq (12.4, 6.2)		3.66[e]	3.70–3.64, m[e]	3.47, m
**6′′′**	1.12, d (6.2)	1.11, d (6.1)	1.12, d (6.2)	1.12, d (6.1)	1.30, d (6.1)	1.28, d (6.1)	1.14, d (6.1)
					2′′-Glucosyl	***2′′-Glucosyl***	
**1′′′′**					4.56, d (7.2)	4.51, d (7.8)	
**2′′′′**					3.23[e]	3.25, m[e]	
**3′′′′**					3.39[e]	3.35, m[e]	
**4′′′′**					3.32[e]	3.31[e]	
**5′′′′**					3.32[e]	3.36, m[e]	
**6′′′′**					3.86[e]	4.49, m[e]	
					3.71[e]	4.18, dd (5.9, 12.0)	
					3′′-Glucosyl	***3′′-Glucosyl***	
**1′′′′′**					4.45, d (7.2)	4.75, d (7.8)	
**2′′′′′**					3.35[e]	3.32, m[e]	
**3′′′′′**					3.47[e]	3.36, m[e]	
**4′′′′′**					3.29[e]	3.28, m[e]	
**5′′′′′**					3.74[e]	3.71, m[e]	
**6′′′′′**					4.53[e]	4.56, m[e]	
					4.42[e]	4.41, m[e]	
					6′′′′′-(E)-p-coumaroyl	***4′′-Glucosyl***	
**1′′′′′′**						4.71, d (7.9)	
**2′′′′′′**					6.24, d (15.9)	3.08, t (8.5)	
**3′′′′′′**					7.53, d (15.9)	3.30, m[e]	
**4′′′′′′**						3.25, m[e]	
**5′′′′′′/9′′′′′′**					7.06, d (8.5)	3.22, m[e]	

**6′′′′′′/8′′′′′′**	6.34, d (8.5)	3.80, m[e]<br>3.62, m[e]
		6''''-(E)-p-coumaroyl
**1′′′′′′′**		
**2′′′′′′′**		6.11, d (15.9)
**3′′′′′′′**		7.42, d (15.9)
**4′′′′′′′**		
**5′′′′′′′/ 9′′′′′′′**		7.24, d (8.5)
**6′′′′′′′/ 8′′′′′′′**		6.65, d (8.5)
**7′′′′′′′**		
		6'''''-(E)-p-coumaroyl
**1′′′′′′′′**		
**2′′′′′′′′**		6.15, d (15.9)
**3′′′′′′′′**		7.45, d (15.9)
**4′′′′′′′′**		
**5′′′′′′′′/ 9′′′′′′′′**		6.95, d (8.6)
**6′′′′′′′′/ 8′′′′′′′′**		6.27, d (8.6)
**7′′′′′′′′**		

[a] Recorded in DMSO-*d6* at 600 MHz; [b] Recorded in DMSO-*d6* at 500 MHz; [c] Recorded in methanol-*d4* at 400 MHz; [d] Recorded in methanol-*d4* at 500 MHz; [e] Overlapped.

Table S2. ^{13}C NMR data for compounds **1**–**6, 8 and 9**.

position	**1**[a]	**2**[b]	**3**[a]	**4**[c]	**5**[d]	**6**[a]	**8**[c]	**9**[b]
	δ_C	δ_C	δ_C	δ_C	δ_C	δ_C	δ_C	δ_C
**2**	156.9	156.8	156.7	161.5	158.5	156.1	158.0	147.5
**3**	133.5	133.5	133.8	135.5	135.4	133.1	136.1	136.1
**4**	177.7	177.4	177.7	179.4	179.2	177.4	179.2	176.1
**5**	160.9	160.1	160.8	163.0	162.5	161.2	162.5	160.4
**6**	98.4	98.4	99.4	100.0	100.6	98.9	100.4	98.8
**7**	161.6	161.6	161.6	166.0	163.4	164.8	163.2	161.4
**8**	94.5	94.5	94.5	94.9	95.3	93.7	95.3	94.3
**9**	156.0	156.0	155.9	158.5	157.7	156.4	157.4	155.8
**10**	105.6	105.7	105.6	105.6	107.5	103.8	107.5	104.7
**1′**	120.7	120.8	120.5	122.7	122.3	120.8	122.3	121.6
**2′**	131.1	131.0	131.6	132.4	131.9	130.8	131.9	129.7
**3′**	115.1	115.2	115.3	116.1	116.6	115.1	116.6	115.5
**4′**	160.3	160.9	160.3	159.4	161.7	160.0	161.4	159.4
**5′**	115.1	115.2	115.3	116.1	116.6	115.1	116.6	115.5
**6′**	131.1	131.0	131.6	132.4	131.9	130.8	131.9	159.4
	3-Galactosyl	***3-Glucosyl***	***3-arabinosyl***	***3-Glucosyl***	***3-Rhamnosyl***	***3-Glucosyl***	***3-Rhamnosyl***	
**1″**	101.5	100.7	101.2	104.6	101.3	100.9	101.5	
**2″**	71.6	74.2	70.7	75.7	80.5	74.2	81.7	
**3″**	73.1	76.4	71.5	78.1	81.7	76.4	80.8	
**4″**	67.9	70.2	66.0	71.4	71.5	69.9	78.3	
**5″**	75.9	77.6	64.2	77.2	71.8	77.5	70.7	
**6″**	60.2	60.9		68.6	17.7	60.8	17.9	
	7-Rhamnosyl	***7-Rhamnosyl***	***7-Rhamnosyl***	***6''-Rhamnosyl***	***7-Rhamnosyl***		***7-Rhamnosyl***	***7-Rhamnosyl***
**1‴**	99.8	99.4	98.3	102.4	99.8		99.7	98.4
**2‴**	70.1	69.9	70.1	72.1	71.7		71.7	70.1
**3‴**	70.3	70.1	70.2	73.9	72.1		72.0	70.3
**4‴**	71.2	71.6	71.6	75.7	73.6		73.6	71.6
**5‴**	69.8	69.8	69.8	69.7	71.1		71.1	69.9
**6‴**	17.9	17.9	17.9	17.9	18.1		18.2	18.0
					2''-Glucosyl		***2''-Glucosyl***	
**1⁗**					105.9		106.0	
**2⁗**					75.3		75.2	
**3⁗**					77.8		78.1	
**4⁗**					77.8		71.8	
**5⁗**					77.9		75.1	
**6⁗**					62.8		64.4	

	3″-Glucosyl	***3″-Glucosyl***
**1′′′′′**	106.1	105.3
**2′′′′′**	75.3	75.3
**3′′′′′**	77.8	77.6
**4′′′′′**	72.4	72.4
**5′′′′′**	75.9	75.9
**6′′′′′**	65.2	65.2
	6′′′′-(E)-p-	***4″-Glucosyl***
**1′′′′′′**	168.9	104.0
**2′′′′′′**	114.8	75.7
**3′′′′′′**	146.8	78.6
**4′′′′′′**	126.6	71.8
**5′′′′′′**	130.5	77.6
**6′′′′′′**	116.3	62.8
**7′′′′′′**	160.7	
		6′′′′-(E)-p-
**1′′′′′′′**		169.0
**2′′′′′′′**		114.8
**3′′′′′′′**		146.7
**4′′′′′′′**		126.9
**5′′′′′′′/9′′′′′′′**		131.0
**6′′′′′′′/8′′′′′′′**		116.5
**7′′′′′′′**		160.9
		6′′′′′-(E)-p-
**1′′′′′′′′**		168.9
**2′′′′′′′′**		114.7
**3′′′′′′′′**		146.5
**4′′′′′′′′**		126.5
**5′′′′′′′′/9′′′′′′′′**		130.4
**6′′′′′′′′/8′′′′′′′′**		116.3
**7′′′′′′′′**		160.6

[a] Recorded in DMSO-*d6* at 150 MHz; [b] Recorded in DMSO-*d6* at 125 MHz; [c] Recorded in methanol-*d4* at 100 MHz; [d] Recorded in methanol-*d4* at 125 MHz.

Table S3. ^{1}H and ^{13}C NMR data for compound 7

position	7[a]	7[b]	No.	7[a]	7[b]
	δ_H, mult (*J* in	δ_C		δ_H, mult (*J* in	δ_C
**2**		157.1	***2″-Glucosyl***		
**3**		134.6	**1″″**	4.32, d (7.8)	106.0
**4**		177.9	**2″″**	3.05, t (8.3)	73.7
**5**		161.0	**3″″**	3.21, m	76.0
**6**	6.40, brs	99.4	**4″″**	3.18, m	69.8
**7**		161.7	**5″″**	3.33, m[c]	73.6
**8**	6.65, brs	94.5	**6″″**	4.23, br d (11.8)	63.0
**9**		155.9		4.12, dd (11.8, 5.5)	
**10**		105.7	***6″″-(E)-p-coumaroyl***		
**1′**		120.1	**1‴″**		166.3
**2′**	7.75, d (8.7)	130.6	**2‴″**	6.14, d (15.9)	113.8
**3′**	6.92, d (8.7)	115.4	**3‴″**	7.41, d (15.9)	144.6
**4′**		160.3	**4‴″**		124.9
**5′**	6.92, d (8.7)	115.4	**5‴″**	7.35, d (8.6)	130.0
**6′**	7.75, d (8.7)	130.6	**6‴″**	6.68, d (8.6)	115.6
3- Rhamnosyl			**7‴″**		159.8
**1″**	5.63, s	100.6	**8‴″**	6.68, d (8.6)	115.6
**2″**	4.14, br s	81.8	**9‴″**	7.35, d (8.6)	130.0
**3″**	3.55, dd (9.6, 3.2)	70.1	5-OH	12.53, s	
**4″**	3.13, m[c]	71.7			
**5″**	3.37, m[c]	70.2			
**6″**	0.92, d (6.2)	17.4			
7-Rhamnosyl					
**1‴**	5.51, s	98.5			
**2‴**	3.84, br s	69.8			
**3‴**	3.63,dd (9.3, 3.2)	70.1			
**4‴**	3.29, m[c]	71.6			
**5‴**	3.44, m[c]	70.1			
**6‴**	1.12, d (6.1)	17.9			

[a] Recorded in DMSO-*d6* at 600 MHz; [b] Recorded in DMSO-*d6* at 150 MHz; [c] Overlapped

Table S4.

Gene (human)	Sequences of primer used	
p16INK4A	Forward	ATATGCCTTCCCCCACTACC
	Reverse	CGTGAGTGCTCACTCCAGAA
p21CIP1	Forward	ATGAAATTCACCCCCTTTCC
	Reverse	CCCTAGGCTGTGCTCACTTC
p53	Forward	GGCCCACTTCACCGTACTAA
	Reverse	GTGGTTTCAAGGCCAGATGT
Glb1	Forward	TTTGACTACCTGCGCTTCCT
	Reverse	AGTCCACCGTGGTGTAGAGG
IL-6	Forward	AGGAGACTTGCCTGGTGAAA
	Reverse	CAGGGGTGGTTATTGCATCT
IL-8	Forward	GTGCAGTTTTGCCAAGGAGT
	Reverse	CTCTGCACCCAGTTTTCCTT
IL-1α	Forward	ATCAGTACCTCACGGCTGCT
	Reverse	TGGGTATCTCAGGCATCTCC
IL-1β	Forward	CTGTCCTGCGTGTTGAAAGA
	Reverse	TTCTGCTTGAGAGGTGCTGA
18s	Forward	CTACCACATCCAAGGAAGCA
	Reverse	TTTTTCGTCACTACCTCCCCG
